# Alterations in Kernel Proteome after Infection with *Fusarium culmorum* in Two Triticale Cultivars with Contrasting Resistance to *Fusarium* Head Blight

**DOI:** 10.3389/fpls.2016.01217

**Published:** 2016-08-17

**Authors:** Dawid Perlikowski, Halina Wiśniewska, Joanna Kaczmarek, Tomasz Góral, Piotr Ochodzki, Michał Kwiatek, Maciej Majka, Adam Augustyniak, Arkadiusz Kosmala

**Affiliations:** ^1^Institute of Plant Genetics, Polish Academy of SciencesPoznan, Poland; ^2^Plant Breeding and Acclimatization Institute—National Research InstituteBlonie, Poland

**Keywords:** amylase, cereals, FHB, *Fusarium*, inhibitors, mycotoxins, proteome

## Abstract

**Highlight:** The level of pathogen alpha-amylase and plant beta-amylase activities could be components of plant-pathogen interaction associated with the resistance of triticale to *Fusarium* head blight.

Triticale was used here as a model to recognize new components of molecular mechanism of resistance to *Fusarium* head blight (FHB) in cereals. *Fusarium*-damaged kernels (FDK) of two lines distinct in levels of resistance to FHB were applied into a proteome profiling using two-dimensional gel electrophoresis (2-DE) to create protein maps and mass spectrometry (MS) to identify the proteins differentially accumulated between the analyzed lines. This proteomic research was supported by a measurement of alpha- and beta-amylase activities, mycotoxin content, and fungal biomass in the analyzed kernels. The 2-DE analysis indicated a total of 23 spots with clear differences in a protein content between the more resistant and more susceptible triticale lines after infection with *Fusarium culmorum*. A majority of the proteins were involved in a cell carbohydrate metabolism, stressing the importance of this protein group in a plant response to *Fusarium* infection. The increased accumulation levels of different isoforms of plant beta-amylase were observed for a more susceptible triticale line after inoculation but these were not supported by a total level of beta-amylase activity, showing the highest value in the control conditions. The more resistant line was characterized by a higher abundance of alpha-amylase inhibitor CM2 subunit and simultaneously a lower activity of alpha-amylase after inoculation. We suggest that the level of pathogen alpha-amylase and plant beta-amylase activities could be components of plant-pathogen interaction associated with the resistance of triticale to FHB.

## Introduction

*Fusarium* head blight (FHB) is a serious plant disease resulting in a significant reduction of kernel quality and yield in small grain cereals. This head infection is caused by several widespread necrotrophic mycotoxigenic fungi of *Fusarium* genus: *F. culmorum* (W.G. Smith.), *F. avenaceum* (Corda ex Fries) Sacc., and *F. graminearum* (Schwabe; Bottalico and Perrone, 2002). The disease symptoms are mainly a result of contamination of *Fusarium*-damaged kernels (FDK) with toxic fungal secondary metabolites (mycotoxins), including e.g., zearalenone (ZEA) and trichothecene B toxins such as deoxynivalenol (DON), nivalenol (NIV), and DON derivatives—3-acetyldexynivalenol (3-AcDON) and 15-acetyldeoxynivalenol (15AcDON; Bottalico and Perrone, 2002; Chakraborty et al., 2006; Buerstmayr et al., 2009; Marin et al., 2013). The *Fusarium* species are harmful mostly to bread wheat (*Triticum aestivum* L.), durum wheat (*T. durum* Desf.), maize (*Zea mays* L.), triticale (× *Triticosecale* Wittm.), oat (*Avena sativa* L.), and rice (*Oryza sativa* L.; Miedaner et al., 2001; Langevin et al., 2004). The selection of cereal genotypes with improved resistance to FHB is a relatively difficult process since the resistance is a quantitative trait governed by genetic factors located both in the host plant and pathogen, and also by environmental conditions, particularly temperature and rainfall, from flowering to the soft-dough-stage of kernel development (Mesterhazy, 1995; Miedaner, 1997; Chełkowski et al., 2000; Snijders, 2004; Cowger et al., 2009). The Quantitative Trait Loci (QTLs) controlling resistance to FHB have been identified in wheat on most chromosomes (Buerstmayr et al., 2009). The QTL with the largest effect was located on 3B chromosome (*Qfhs.ndsu-3BS*) in the Chinese wheat cultivar Sumai 3 and it was shown to be associated with the FHB resistance gene *Fhb1* (Cuthbert et al., 2006). The other QTLs were also mapped and named e.g., *Fhb2, Fhb4, Fhb5* (Cuthbert et al., 2007; Xue et al., 2010, 2011), however, these chromosomal regions confer only partial resistance (Bai and Shaner, 2004). To date, the resistance to FHB was classified into five types including resistance against initial infection (I type), resistance to *Fusarium* spread within the spike (II type), resistance to kernel infection (III type), tolerance to FHB and toxins (IV type), and resistance to toxin accumulation (chemical modification or synthesis inhibition; V type; Mesterhazy, 1995; Boutigny et al., 2008; Foroud and Eudes, 2009).

Triticale has been obtained by crossing of hexaploid or tetraploid wheat as a female parent with diploid rye (*Secale cereale* L.) as a male parent (Cichy et al., 2002; Oettler, 2005). This intergeneric, man-made hybrid combines the complementary traits of both parental species, high yielding capacity of wheat and stress tolerance of rye, however, there are only limited reports concerning resistance to FHB in triticale (Miedaner et al., 2001, 2004; Góral et al., 2002; Góral and Ochodzki, 2007). Although, this species is thought to be less susceptible to FHB, compared to wheat and more susceptible, compared to rye (Arseniuk et al., 1993; Langevin et al., 2004), the other studies revealed that susceptibility to FHB in triticale may be equal to that observed in wheat or even exceeds it (Miedaner et al., 2001; Langevin et al., 2004; Comeau et al., 2008; Veitch et al., 2008). Recently, the report on QTLs associated with FHB resistance in triticale has been published (Kalih et al., 2015) but the molecular nature of this trait still remains unrecognized in detail. Taking into account a genomic constitution of triticale and its origin, this hybrid could be a good model for the other cereals to recognize new crucial components of resistance to FHB not revealed to date in the parental species.

Proteome profiling to investigate mechanisms of resistance to FHB in cereals has been shown before e.g., in wheat (e.g., Zhou et al., 2005, 2006; Eggert et al., 2011) and barley (*Hordeum vulgare* L.; Yang et al., 2010a,b; Eggert and Pawelzik, 2011) infected with *F. culmorum* or/and *F. graminearum* (for review: Yang et al., 2013). Also our earlier work on winter wheat infected with *F. culmorum* could be treated as a good example of such research (Perlikowski et al., 2014). The aspect of proteomic approach to recognize in cereals markers associated with their resistance to the selected biotic stresses has been recently reviewed by Kosová et al. (2014).

Here, we demonstrate the first proteomic research for triticale, including: (1) the analysis of protein abundance in the FDK of two lines, more susceptible and more resistant to FHB using two-dimensional gel electrophoresis (2-DE) and (2) mass spectrometry (MS) to identify differentially accumulated proteins. The proteome screening was followed by the alpha- and beta-amylase activity assays to reveal a potential involvement of these enzymes into the resistance of triticale to FHB. This proteomic research was supported by the evaluation of fungal biomass as well as mycotoxin content in the analyzed kernels.

## Materials and methods

### Field experiments

The scientific approach for field experiments was similar to that described previously for wheat by Perlikowski et al. (2014). The plant materials for the research reported here were two lines of hexaploid triticale (× *Triticosecale* Wittm.)—DS 9, a line with a relatively high level of resistance to FHB (RL) and DANKO 1, a line with a relatively high level of susceptibility (SL), both developed by Danko Plant Breeding Ltd., Co. (Poland). The resistance levels of the analyzed triticale lines were estimated in 2014, in two locations under the field conditions: Cerekwica (western Poland; GPS coordinates: N 52.521012, E 16.692005) characterized by poor, sandy-clay soil and Radzikow (central Poland; GPS coordinates: N 52.211754, E 20.631954) with rich sandy-clay soil. The rainfalls and mean temperature during the experiments performed in Cerekwica and Radzikow, are presented in Table S1. The experiments in both locations were carried out according to the same design. The experimental field in each location consisted of four plots for each tested line. The seeds were sown in plots of 1 m^2^ size with the sowing rate 300 seeds (September, 2013). The fungal material for inoculation was a mixture of three isolates of *F. culmorum* (W.G.Sacc.): KF 846 (DON chemotype) and KF 350 (NIV chemotype) derived from the collection of Institute of Plant Genetics, Polish Academy of Sciences (Poznan, Poland), and ZFR 112, producing zearalenone (ZEA), derived from the collection of Plant Breeding and Acclimatization Institute—National Research Institute (Radzikow, Poland; Snijders and Perkowski, 1990; Wiśniewska and Kowalczyk, 2005). In the case of each analyzed line, one plot, used as a control, was not treated with *Fusarium* isolates, and the flowering triticale heads at three other field plots were sprayed with the spore suspension at a rate of ~100 ml m^−2^ (May, 2014). The developmental stage of the heads was 65, in commonly used BBCH scale, which means: full flowering and 50% of anthers mature. The concentration of conidia was adjusted to 5 × 10^4^ ml^−1^. A micro-irrigation was applied during 2 days after inoculation, and after next 15 days, a progress of the disease was evaluated visually. The percentage of heads infected per plot and percentage of head infection were determined. The FHB index (FHBi), associated with the resistance type I and II (Mesterhazy, 1995), was calculated, separately for each line and location, according to the formula:

$$\begin{array}{l} \text{FHBi }(\%)=(\%\text{ of head infection }\times \;\%\text{ of heads infected per}\\ \text{ plot})/100 \end{array}$$

In August 2014, 20 randomly selected heads from each experimental plot for the RL and the SL, were threshed manually. Kernels were visually scored and divided into two categories: Healthy-looking kernels (HLK) and FDK. Kernel weight [g] and number were estimated. Percentage of FDK (% FDK) was calculated as a proportion of infected kernels per sample. Mean values and standard deviations of this parameter calculated on the base of three inoculated plots were shown in the paper, separately for each location and line. The % FDK is associated with the type III FHB resistance (Mesterhazy, 1995). Total kernel number and weight per head from 20 randomly selected heads from one control plot, separately for each line and location, were also estimated.

Analysis of variance in FHBi, FDK (weight and number), a total kernel number per head and a total kernel weight per head was performed using the ANOVA procedure of XLSTAT (Microsoft® Excel 2010/XLSTAT©-Ecology Version 2016.02.28540, Addinsoft, Inc., Brooklyn, NY, USA). Multiple comparisons of means of lines in locations were performed using Fisher (LSD) test.

### Mycotoxin analysis

The accumulation level of trichothecene B in the kernels of the RL and the SL triticale lines was evaluated. This analysis involves deoxynivalenol, nivalenol, 3-acetyldexynivalenol, and 15-acetyldeoxynivalenol. Additionally, the level of zearalenone was also estimated.

#### Trichthecenes B

The amount of 5 g of the ground sample was placed in a conical 50 ml Falcon centrifuge tube and then 25 ml of the solvent (acetonitrile-water 84:16 v/v) was added. The sample was extracted for 2 h on a shaker and then centrifuged (1620 g, 5 min). A clear extract volume of 6 ml was purified on Trich 227 SPE column (Multisep® 227 Trich+, Romer Labs, ®Inc. Union, Mo, USA) and 4 ml of purified extract was transferred to vial, 100 μl of internal standard (chloralose 10 μg/ml) was added and then evaporated to dryness in heating block under stream of nitrogen in 40°C. To the dried extract in 4 ml vial 75 μl of Sylon BTZ [BSA (N,O-Bis(trimethylsilyl)acetamide]:TMCS (Chlorotrimethylsilane):TMSI [1-(Trimethylsilyl) imidazole), 3:2:3] (Supelco, Bellefonte, USA) was added and heated for 30 min in 60°C in oven. After cooling sample was dissolved in 1 ml of isooctane, the excess of silylating agent was removed by washing twice with 1 ml of distilled water and organic layer was transferred to 2 ml autosampler vial. Chromatographic analysis was conducted on SRI 8610C gas chromatograph (SRI Instruments, Torrance, USA) equipped with electron-capture detector (ECD) and capillary column BGB-5MS, 30 m, 0.25 mm, 0.2 μm film thickness. Hydrogen was the carrier gas at constant pressure and nitrogen was the make-up gas, constant flow 60 ml/min was applied. Injector temperature was established at 250°C and detector temperature at 320°C. A sample volume of 1 μl was injected in splitless mode. The initial temperature of column was 170°C, held for 2 min, then increased by 5°C/min to 245°C, held for 2 min, increased again by 25°C/min to 300°C, and held finally for 7 min. The content of each toxin was expressed as toxin weight [mg] per kernel weight [kg].

#### Zearalenone

A 5 g of the ground sample was placed in a conical 50 ml Falcon centrifuge tube and then 25 ml of the solvent (methanol-water 70:30 v/v) was added. The sample was extracted for 1 h on a shaker and then centrifuged (1620 g, 5 min). The obtain extract was analyzed with ELISA method according to the procedure described by RomerLabs, Agraquant (http://shop.romerlabs.com/en/AgraQuant-ELISA/AgraQuant-Mycotoxins). The content of zearalenone was expressed as toxin weight [mg] per kernel weight [kg].

### Evaluation of pathogen biomass in the kernels

To produce mycelium KF 846 isolate of *F. culmorum* was maintained during 5 days on Potato Dextrose Agar plates (PDA, Sigma, UK) with streptomycin (100 μg/ml) at room temperature. Fungal tissue was lyophilized, frozen in liquid nitrogen, and ground to a fine powder. Further, it was mixed with *F. culmorum*-free ground triticale kernels in 10-fold dilution series from 10 to 0.001 mg/g (Horevaj et al., 2011). DNA was extracted from each serial dilution and 0.5 g triticale kernels of the RL and SL lines using the CTAB (cetyltrimethyl ammonium bromide). The samples were suspended in 650 μl CTAB and incubated at 65°C for 20 min. The volume of 500 μL CHCl_3_ was added and centrifuged at 12 879 g for 15 min. DNA was precipitated with 65 μl 3M sodium acetate, pH 5.4, and two volumes of ice cold 99.8% ethanol. The tubes were stored at −20°C overnight and centrifuged at 17 530 g for 5 min. The pellets were washed with 70% ethanol, centrifuged at 17,530 g for 5 min and fully dissolved in 100 μl of TE buffer.

The *real-time* PCR was performed in 10 μl containing 7.5 μl AmpliQ *Real-Time* PCR Opti Probe Kit (Novazym, Poznań, Poland), 100 nM of FAM-labeled probe and 300 nM of forward and reverse *F. culmorum* primers (Waalwijk et al., 2004). Thermal cycling parameters for a quantitative fungal DNA detection were: 95°C for 2 min followed by 40 cycles of 95°C for 15 s and 60°C for 30 s. Nuclease-free water was used as the no-template control. A standard curve was generated by plotting the *C*_*t*_ value for each sample of standard series of the amount of fungal biomass (10–0.001 mg/g). All the samples were tested in triplicate.

Analysis of variance in *Fusarium* biomass was performed using the ANOVA procedure of XLSTAT (Microsoft® Excel 2010/XLSTAT©-Ecology Version 2016.02.28540, Addinsoft, Inc., Brooklyn, NY, USA). Multiple comparisons of means of lines in locations were performed using Fisher (LSD) test.

### Proteome profiling and identification of differentially accumulated proteins

**Figure 1:**
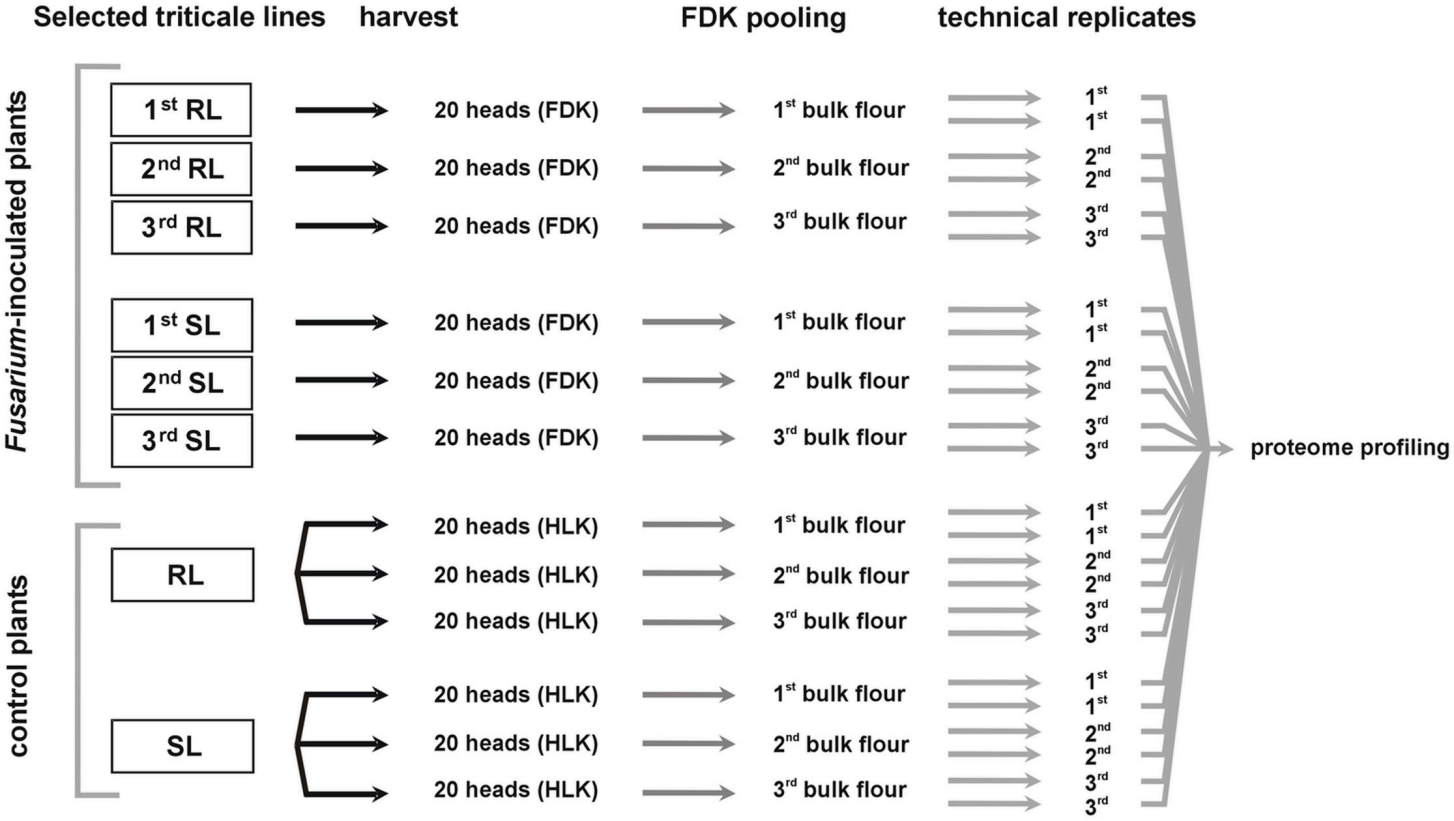
**Diagram demonstrating a workflow of sample preparation for proteome analysis**. FDK, *Fusarium*-damaged kernels; HLK, healthy-looking kernels; RL, line of triticale more resistant to *Fusarium* head blight; SL, line of triticale more susceptible to *Fusarium* head blight.

The plant materials derived from one location (Cerekwica) were used for further molecular research. The FDK derived from 20 heads were pooled, separately for each inoculated plot, giving three separate pooled samples (bulk flour) for each analyzed line, the RL and the SL. The kernels derived from 20 heads of the control plot were also pooled for each analyzed line. The pooled samples (bulk flour) were used for proteomic research—each one in two technical replicates. A diagram outlining the workflow of sample preparation for proteome analysis is shown in the Figure 1.

The proteomic protocol used, including 2-DE and MS to identify differentially accumulated kernel proteins between the RL and SL of triticale, was the same as that described in detail by Perlikowski et al. (2014). Proteins were extracted as described by Hurkman and Tanaka (1986) and their concentration in samples estimated using 2-D Quant Kit (GE Healthcare, Buckinghamshire, UK). In isoelectrofocusing (IEF), strip gels with linear pH range 4–7 (24 cm) were used to focus 500 μg of proteins extracted from 25 mg of triticale flour. This pH range was selected on the basis of our earlier work on wheat and our preliminary proteome screening in triticale. It was shown to be a good compromise between gel quality, its resolution, and spot numbers. In the second dimension (sodium dodecyl sulfate-polyacrylamide gel electrophoresis) the proteins were separated using 13% polyacrylamide gels (1.5 × 255 × 196 mm). The gels were stained with colloidal coomassie brilliant blue G-250 as described by Neuhoff et al. (1988), scanned by Image scanner III (GE Healthcare, Buckinghamshire, UK) and subjected to Labscan 6.0 program (GE Healthcare, Buckinghamshire, UK) processing. The image analysis was performed with Image Master 2-D *Platinum* software (GE Healthcare, Buckinghamshire, UK). The abundance of each protein spot was normalized as a relative volume (% Vol) and calculated as a ratio of the volume of particular spot to the total volume of all the spots present on the gel. The spot had to be detected in all the replicates to be consider as “present.” The significance of the differences was assessed using Kolmogorov–Smirnov Test (three biological replicates, each one with two technical replicates used as means). The protein spots which showed at least two fold differences in protein content between two analyzed lines were analyzed by liquid chromatography coupled to the Orbitrap Velos type mass spectrometer (Thermo Fisher, Waltham, MA, USA), working in the regime of data dependent MS to MS/MS switch.

The MS analysis was performed in the Laboratory of Mass Spectrometry, Institute of Biochemistry and Biophysics, Polish Academy of Sciences (Warsaw, Poland) as shown earlier by Kosmala et al. (2012) and Perlikowski et al. (2014). The data was analyzed with Mascot Distiller software (version 2.3, MatrixScience London, UK) with standard settings for the Orbitrap low resolution measurements (available at http://www.matrixscience.com/distiller.html) to extract MS/MS peak-lists from the raw files. The obtained fragmentation spectra were matched to the National Center for Biotechnology Information (NCBI) non-redundant database (57412064 sequences; 20591031683 residues), with a *Viridiplantae* filter (2874321 sequences) using the Mascot search engine (Mascot Daemon v. 2.3.0, Mascot Server v. 2.4.0, MatrixScience, London, UK). The search parameters were the same as those described in details by Perlikowski et al. (2014). The MS proteomics data has been deposited to the ProteomeXchange Consortium via the PRIDE (https://www.ebi.ac.uk/pride/archive/) partner repository with the dataset identifier PXD004464.

### Alpha- and beta-amylase activity assays

Alpha-amylase activity in triticale kernels was evaluated using the Ceralpha α-Amylase Assay Kit (Megazyme International Ireland Inc., Bray, Ireland) as described in our earlier work on wheat (Perlikowski et al., 2014).

Beta-amylase activity was tested using the “Betamyl-3® method” Assay Kit (Megazyme International Ireland Ltd., Bray, Ireland). One unit of activity was defined as the amount of enzyme, in the presence of excess thermostable β-glucosidase, required to release one micromole of *p*-nitrophenol from *p*-nitrophenyl-β-D-maltotrioside in 1 min under the defined assay conditions.

Three biological and two technical replicates were used (Figure 1). Each technical replicate contain a flour in an amount of 0.5 g. The enzyme activity was shown in Ceralpha Units (CU) per gram of flour and the significance of differences between the RL and SL was assessed using ANOVA (*p* ≤ 0.05).

## Results and discussion

### Field experiments and mycotoxin analysis

The two analyzed triticale lines were revealed to have significantly different levels of resistance to FHB. This phenomenon was manifested by the values of FHBi in Cerekwica and % FDK in two locations (Table 1). These triticale lines were also significantly different with respect to a mycotoxin content (Table 2). In the control conditions the resistant line showed a higher yield level, compared to the susceptible line (Table 1). The differences revealed for both locations could be a result of different soil quality and weather conditions (Table S1).

**Table 1:** **The components of the resistance to *Fusarium* head blight in the more resistant (RL) and more susceptible (SL) triticale lines and their yields under control conditions**.

**Triticale line**	**Location**	**Conditions after inoculation**						**Control conditions**	
		**FHBi**	**% FDK (weight [g])**	**% FDK (number)**	**Total kernel number/head**	**Total kernel weight [g]/head**	***F. culmorum* biomass [mg/g]**	**Total kernel number/head**	**Total kernel weight [g]/head**
RL	Cerekwica	14 ± 2.83b	9.02 ± 1.10c	14.99 ± 2.43c	39.65 ± 2.76a	1.78 ± 0.08b	2.60 ± 0.36b	74.45	3.02
SL		24 ± 5.66a	66.55 ± 7.29a	82.43 ± 3.83a	36.81 ± 2.80b	0.82 ± 0.16c	5.26 ± 0.12a	51.65	2.71
RL	Radzikow	8.1 ± 1.15b	3.94 ± 1.19d	5.03 ± 1.51d	50.98 ± 1.48a	2.55 ± 0.17a	0.76 ± 0.12d	59.05	3.05
SL		12.0 ± 4.58b	39.36 ± 1.63b	49.63 ± 1.16b	42.83 ± 4.33a	1.72 ± 0.13b	1.97 ± 0.30c	49.25	2.60

*FHBi, Fusarium head blight index; FDK, Fusarium-damaged kernels; RL, more resistant line; SL, more susceptible line; mean values and standard deviations of each parameter calculated after inoculation (three plots) and data from one plot calculated for the control conditions, are shown. Values marked with the same letter are not significantly different according to Fisher (LSD) test at p = 0.05*.

**Table 2:** **The toxin content in the kernels of the more resistant (RL) and more susceptible (SL) triticale lines**.

**Triticale line**	**Location**	**DON [mg/kg]**	**15AcDON [mg/kg]**	**NIV [mg/kg]**	**ZEA [mg/kg]**
RL	Cerekwica	9.800	0.654	6.537	0.248
SL		113.920	31.403	27.245	4.349
RL	Radzikow	0.540	0.080	0	0.010
SL		9.325	0.792	0	0.359

*RL, more resistant line; SL, more susceptible line*.

### Pathogen biomass in the kernels

The differences in the levels of resistance to FHB between the triticale lines were also manifested by the values of fungal biomass in the analyzed kernels. Consistently with the visual assessments of FHB, the line with a higher disease index (SL) revealed simultaneously a higher amount of fungal biomass assessed by qPCR. No *F. culmorum* tissue was detected in the kernels collected in the control conditions (Table 1).

### Proteome profiles and identities of differentially accumulated proteins

**Figure 2:**
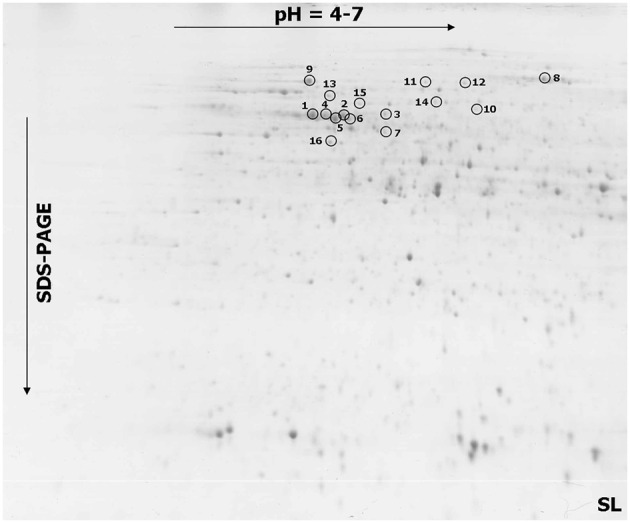
**One representative 2-DE protein map of triticale kernel after *Fusarium culmorum* infection (*Fusarium*-damaged kernels) for the line more susceptible (SL) to *Fusarium* head blight**. The spots with differentially accumulated proteins (1–16) identified in the SL, are circled with a solid line.

**Figure 3:**
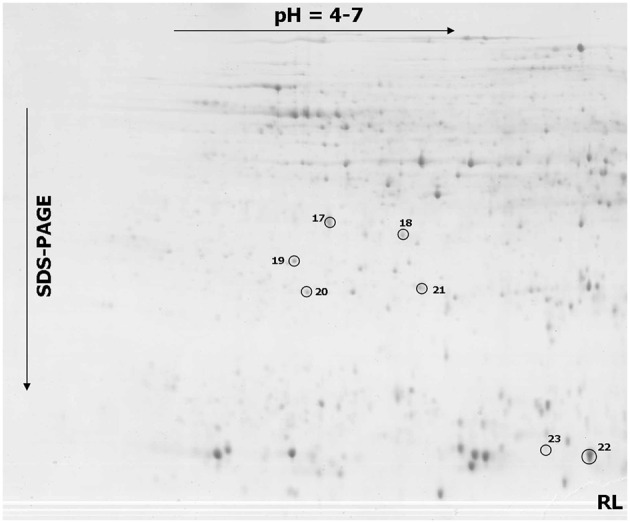
**One representative 2-DE protein map of triticale kernel after *Fusarium culmorum* infection (*Fusarium*-damaged kernels) for the line more resistant (RL) to *Fusarium* head blight**. The spots with differentially accumulated proteins (17–23) identified in the RL, are circled with a solid line.

The comparative analyses indicated a total of 23 spots that showed significant differences in a protein abundance between the more resistant and more susceptible triticale lines after infection (Figures 2, 3 and Figures S1, S2), including 16 spots with a significantly higher protein abundance in the more susceptible line (spots no. 1–16) and seven spots with a significantly higher abundance in the more resistant line (spot no. 17–23). All the selected protein spots were subjected to MS identification (Table 3) and in all the cases these selections were identified as homologs of proteins from related plant species (Table 3). A majority of the identified proteins (according to UniProt categories; www.uniprot.org) were involved in a cell carbohydrate metabolism with 10 proteins highly accumulated in the SL (spots no. 1, 2, 4, 5, 6, 7, 8, 9, 10, and 15) and two in the RL (spots no. 20 and 22), stressing the importance of this protein group in plant response to *Fusarium* inoculation. The relevance of the regulation of carbohydrate metabolism for plant-pathogen integrations was postulated earlier (for review: Berger et al., 2007). The other identified proteins were involved in an amino acid metabolism (spots no. 3, 11, and 12), a protein biosynthesis and folding (spots no. 13 and 21), a nutrient storage (spot no. 18), a cell redox homeostasis (spot no. 19), a detoxification system (spot no. 17), a mitochondrial electron transport (spot no. 14), and a RNA processing (spot no. 16). The earlier studies performed on other cereal species, revealed also that fungal infection is followed by alterations in plant proteome. In hexaploid wheat infected with *F. graminearum*, 15 proteins were induced or up-regulated. Among them, the antioxidants, such as superoxidase dismutase and glutathione S-transferase, as well as pathogenesis-related proteins, such as beta-1, 3 glucanase, were detected (Zhou et al., 2005). In *T. dicoccum* infected with the same fungal species, 10 proteins changed abundance, including globulin-2 (3) and beta-amylase, identified also in our present study (Eggert et al., 2011). The potential involvement of amylases into plant-fungal interactions and mechanisms of resistance to FHB was suggested earlier e.g., for *H. vulgare* (Yang et al., 2010b) and *Triticum* species (Packa et al., 2013; Perlikowski et al., 2014). The relationship between amylase activities and resistance to FHB exists also, to a high probability, in triticale, as indicated by our first study regarding this matter.

**Table 3:** **The results of MS analysis performed on the spots that showed at least a 2.0 ratio in protein abundance between the more resistant and more susceptible triticale lines**.

**Spot no^a^**	**Accession^b^**	**Identified protein^c^**	**Score^d^**	**Coverage (%)^e^**	**No. of peptide matched**	**Maximum ion score**	**Theor. MW [kDa]/pI^f^**	**Exp. MW [kDa]/pI^g^**
1	EMS68884	beta-amylase [*Triticum urartu*]	1572	44	19	131	59/5.34	59/5.52
2	EMS68884	beta-amylase [*T. urartu*]	1430	35	16	130	59/5.34	59/5.64
3	EMT05050	2-isopropylmalate synthase A [*Aegilops tauschii*]	1843	49	22	132	68/6.31	61/5.82
4	EMT06941	beta-amylase [*A. tauschii*]	1866	49	21	133	60/5.07	59/5.58
5	EMS68884	beta-amylase [*T. urartu*]	2003	53	22	162	59/5.34	58/5.61
6	EMT06941	beta-amylase [*A. tauschii*]	1610	44	19	130	60/5.07	58/5.67
7	AAF61173	small subunit ADP glucose pyrophosphorylase [*T. aestivum*]	1566	48	19	146	52/5.53	51/5.82
8	EMS65561	sucrose synthase 2 [*T. urartu*]	2594	43	32	133	93/6.01	91/6.49
9	EMT06941	beta-amylase [*A. tauschii*]	678	19	9	98	60/5.07	84/5.50
10	EMS58307	pyrophosphate–fructose 6-phosphate 1-phosphotransferase subunit beta [*T. urartu*]	1374	28	18	128	61/7.01	61/6.27
11	EMS51950	5-methyltetrahydropteroyltriglutamate-homocysteine methyltransferase [*T. urartu*]	2078	34	22	161	85/5.74	86/6.02
12	EMS51950	5-methyltetrahydropteroyltriglutamate-homocysteine methyltransferase [*T. urartu*]	2325	36	25	176	85/5.74	85/6.17
13	EMS51416	heat shock 70 kDa protein, mitochondrial [*T. urartu*]	2720	45	29	162	76/6.16	73/5.58
14	EMS46614	succinate dehydrogenase [ubiquinone] flavoprotein subunit, mitochondrial [*T. urartu*]	1292	31	14	140	66/5.98	68/6.08
15	EMT28450	2,3-bisphosphoglycerate-independent phosphoglycerate mutase [*A. tauschii*]	2240	49	27	154	66/5.93	67/5.72
16	AFW69337	putative DEAD-box ATP-dependent RNA helicase family protein [*Zea mays*]	1536	42	18	126	47/5.29	49/5.60
17	EMT08036	lactoylglutathione lyase [*A. tauschii*]	1371	57	17	122	33/5.43	35/5.61
18	ACJ65514	globulin 3 [*T. aestivum*]	402	5	5	136	67/7.78	34/5.84
19	AAK49425	protein disulfide isomerase 3 precursor [*T. aestivum*]	716	20	10	109	57/4.96	32/5.48
20	P46226	triosephosphate isomerase, cytosolic [*S. cereale*]	976	38	9	123	27/5.24	27/5.53
21	AAZ95171	eukaryotic translation initiation factor 5A1 [*T. aestivum*]	338	23	5	106	18/5.70	28/5.98
22	CBA13559	putative alpha-amylase inhibitor CM2, partial [*T. aestivum*]	536	52	5	158	14/5.83	14/6.75
23	EMS64128	hypothetical protein TRIUR3_21203 [*T. urartu*]	474	46	6	121	16/6.3	15/6.57

a *Spot numbering was the same as in Figures 2, 3*.

b *Database accession (according to NCBInr) of a homologous protein*.

c *Homologous protein and organism from which it originates*.

d *Mascot MudPIT (Multidimensional Protein Identification Technology) score*.

e *Amino acid sequence coverage for the identified proteins; amino acid sequences for the proteins were shown in Figure S3*.

f *Theoretical molecular weight and isoelectric point revealed by Mascot software*.

g *Experimental molecular weight and isoelectric point calculated based on 2-D protein maps*.

### Alpha- and beta-amylase activity could be a component of the susceptibility to FHB in triticale

**Figure 4:**
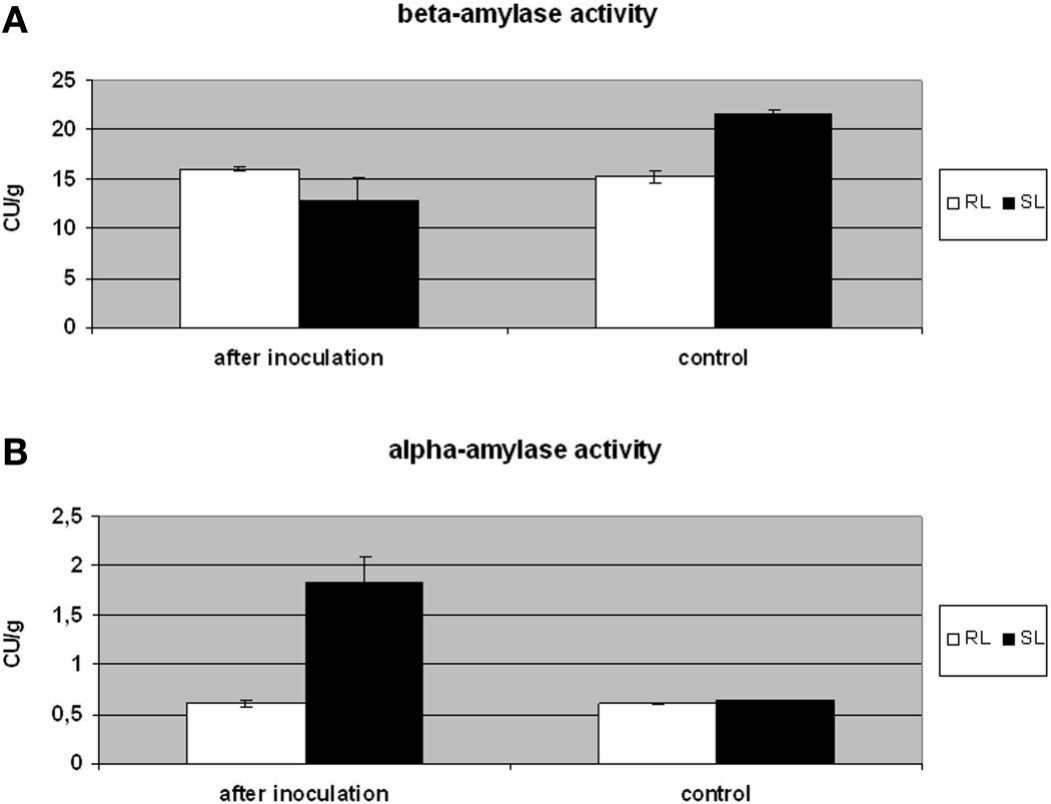
**Comparison of beta- amylase (A) and alpha-amylase (B) activity in the kernels of triticale SL (line more susceptible to *Fusarium* head blight) and RL (line more resistant to *Fusarium* head blight) after *Fusarium culmorum* infection *(Fusarium*-damaged kernels) and in control conditions**. The enzyme activity was expressed in Ceralpha Units (CU) per gram of flour. The means of three biological replicates and standard deviation bars are shown. The significance of differences between the RL and SL was assessed using ANOVA (*p* ≤ 0.05).

Natural kernel sprouting requires starch decomposition by plant alpha- and beta-amylases (Lunn et al., 2001). However, *Fusarium* pathogens can also use plant or their own hydrolytic enzymes to colonize kernels (Wang et al., 2005). The triticale beta-amylase, which significantly accumulated in the SL samples, was identified in six spots (spots no. 1–2, 4–6, and 9; Figure S2). The isoform present in spots no. 1, 2, and 5 possesses 94% of amino acid sequence identity with the isoform present in spots no. 4, 6, and 9 (Figure S3). The particular proteins identified as amylase isoforms might vary in post-translation modifications, resulting in different isoelectric points and molecular weights affecting spot positions in the 2-D gels (Figures 2, 3 and Table 3). Here, the increased accumulation levels of different isoforms of beta-amylase were observed for the SL triticale line after inoculation (spots no. 1–2, 4–6, and 9) and also in the control conditions (spots no. 6 and 9). However, these elevated accumulation levels were not followed by a total level of activity of that enzyme observed after inoculation. The activity was shown to be a slightly higher in the RL and, furthermore, comparable to the level observed in the control conditions in that line (Figure 4A). Thus, the level of beta-amylase activity did not change after infection with *Fusarium* in the resistant line. On the other hand, the susceptible line in the control conditions revealed a higher activity level of beta-amylase, compared to the RL (Figure 4A), indicating simultaneously a higher level of starch degradation in this line before inoculation. After inoculation the enzyme activity was significantly reduced. This phenomenon could explain to a certain degree a higher level of susceptibility of DANKO 1 line to *F. culmorum* before infection. Interestingly, as mentioned earlier, two isoforms of beta-amylase (spots no. 6 and 9) showed also higher accumulation levels in the SL in the control conditions. A discrepancy between accumulation and activity levels of beta-amylase after inoculation has not been explained here in detail. However, it is highly probable that the accumulated isoforms can play also a role in the other metabolic pathways.

It was shown earlier that in field conditions the inoculation with *F. culmorum* led to an increased alpha-amylase activity in kernels of *T. monococum, T. dicoccum*, and *T. aestivum* (Packa et al., 2013; Perlikowski et al., 2014). Our earlier work on winter wheat showed that the alpha-amylase activity level was lower in the wheat RL, both in the control conditions and after inoculation, and we suggested that this could be due to the presence of monomeric alpha-amylase and dimeric alpha-amylase inhibitors, accumulated to a higher degree in the wheat line with a higher resistance to FHB (Perlikowski et al., 2014). Here, a higher accumulation level of alpha-amylase inhibitor CM2 subunit (spot no. 22) both in the control conditions and after inoculation was also detected in the RL triticale line (Figure S2). These results were supported by a lower level of alpha-amylase activity after *Fusarium* inoculation in the RL (Figure 4B). Tetrameric, CM amylase inhibitors are generally composed of one CM1 or CM2 subunit, plus one CM16 or CM17 subunit and plus two CM3 subunits. The inhibitory activity of the protein is dependent on the combination of subunits, however, it was proved to be active against different pathogen alpha-amylases, but not against cereal enzymes (Altenbach et al., 2011). Plant and pathogen alpha-amylase activities were not precisely distinguished here, however, it is highly probable that this amylase activity had its source in both organisms. In the control conditions, when the kernels lacked pathogen biomass (Table 1), this activity could have been fully an attribute of triticale. On the other hand, after inoculation, it could have been mostly an attribute of *Fusarium*, especially in the SL. The resistant line revealed after inoculation the level of alpha-amylase activity comparable to that observed in the control conditions (Figure 4B). Thus, we suggest here that the increased accumulation and activity levels of that enzyme after inoculation in the SL could be a result of its production by *Fusarium* and this phenomenon simultaneously could improve pathogen propagation. This might be possible to a certain degree because of less effective alpha-amylase inhibitory system present in the SL.

## Conclusions

Although, the infection of triticale with *F. culmorum* resulted in abundance alterations of different proteins, the group associated with carbohydrate metabolism was revealed to be the most numerous. The majority of identified proteins in that group were the components of cell amylase machinery, including plant alpha-amylase inhibitor and isoforms of plant beta-amylases. The plant alpha-amylase inhibitors were proved earlier to be the important components of the active resistance of plants to necrotrophic pathogens (Svensson et al., 2004). Thus, the inhibition of pathogen alpha-amylase activity, observed in our study, could also prevent infection progress in the analyzed here more resistant triticale line, however, next experiments are required using more cultivars, locations and different environmental conditions. The similar phenomenon was observed earlier in winter wheat (Perlikowski et al., 2014). Moreover, the activity level of plant beta-amylase before *Fusarium* inoculation in triticale could be responsible, at least partially, for a different susceptibility of the analyzed lines to a pathogen infection. Further research is required to go deeper into the mechanisms of cereals' resistance to FHB, including the involvement of particular amylases into that process. The next experiments should involve work on the other cereals, including rye and other proteomic methods based on fluorescent dyes and gel free protein quantifications.

## Author contributions

AK and HW conceived and designed the experiments. DP, HW, JK, TG, PO, AA, and AK performed the experiments. DP, HW, JK, TG, MK, MM, and AK analyzed the data. HW, TG, JK, PO, and AK contributed reagents/materials/analysis tools. AK wrote the first version of the manuscript. All the authors read and approved the manuscript.

### Conflict of interest statement

The authors declare that the research was conducted in the absence of any commercial or financial relationships that could be construed as a potential conflict of interest.
