# Supplementary material for: Alterations in Kernel Proteome after Infection with Fusarium culmorum in Two Triticale Cultivars with Contrasting Resistance to Fusarium Head Blight
Source: Front Plant Sci. 2016 Aug 17;7:1217. doi: 10.3389/fpls.2016.01217 (PMC4987376; doi:10.3389/fpls.2016.01217)
Supplement: Supplementary file 1 [file Table1.docx]

**Table S1.** Meteorological conditions (sum of rainfalls and mean temperature) during the experiments performed in Cerekwica and Radzikow in 2014.

| Location | Rainfall  [mm] | | | Mean temperature  [°C] | | |
| --- | --- | --- | --- | --- | --- | --- |
|  | May 20-31^*^ | June | July | May 20-31^*^ | June | July |
| Cerekwica | 62.0 | 28.2 | 61.4 | 18.1 | 16.9 | 22.2 |
| Radzikow | 46.0 | 61.0 | 78.4 | 18.2 | 16.3 | 21.6 |

^*^flowering period of triticale
