# Supplementary material for: Alterations in Kernel Proteome after Infection with Fusarium culmorum in Two Triticale Cultivars with Contrasting Resistance to Fusarium Head Blight
Source: Front Plant Sci. 2016 Aug 17;7:1217. doi: 10.3389/fpls.2016.01217 (PMC4987376; doi:10.3389/fpls.2016.01217)
Supplement: Supplementary file 2 [file Image1.pdf]

“Alterations in kernel proteome after infection with *Fusarium culmorum* in two triticale cultivars with contrasting resistance to *Fusarium* head blight”

Dawid Perlikowski[1#], Halina Wiśniewska[1#], Joanna Kaczmarek[1], Tomasz Góral[2], Piotr Ochodzki[2], Michał Kwiatek[1], Maciej Majka[1], Adam Augustyniak[1], Arkadiusz Kosmala[1*]

Figure S1 The quantitative calculations and statistics to compare the selected kernel protein abundance after *Fusarium culmorum* infection in the triticale SL (line more susceptible to *Fusarium* head blight) and the RL (line more resistant to *Fusarium* head blight). Spot numbering is the same as in Fig. 2 and 3. The means of spot %Vol (normalized volume) for three biological replicates were calculated. Two technical replicates for each biological replicate were used as means. The significance of differences between the RL and SL after infection was assessed using Kolmogorov-Smirnov Test.

Composed data Kolmogorov-Smirnov Test

	Samples						Max Neg	Max Pos	p-value	Mean	Mean	Std.Dev.	Std.Dev.	Valid N	Valid N
		**SL**			**RL**		Differnc	Differnc		SL	RL	SL	RL	SL	RL
**Spot 1**	2,4200	2,7300	2,5800	0,8900	0,9700	0,9150	0	1	p < .10	2,5767	0,9250	0,1550	0,0409	3	3
**Spot 2**	2,0500	1,9000	2,0000	0,7900	0,6700	0,7300	0	1	p < .10	1,9833	0,7300	0,0764	0,0600	3	3
**Spot 3**	0,2500	0,2950	0,2800	0,1200	0,1400	0,1300	0	1	p < .10	0,2750	0,1300	0,0229	0,0100	3	3
**Spot 4**	1,1000	1,3000	1,2000	0,4300	0,2900	0,3600	0	1	p < .10	1,2000	0,3600	0,1000	0,0700	3	3
**Spot 5**	1,5000	1,3000	1,4000	0,4300	0,3300	0,3800	0	1	p < .10	1,4000	0,3800	0,1000	0,0500	3	3
**Spot 6**	0,5600	0,6000	0,5800	0,2300	0,3300	0,2800	0	1	p < .10	0,5800	0,2800	0,0200	0,0500	3	3
**Spot 7**	0,2300	0,2400	0,2400	0,0900	0,0880	0,0900	0	1	p < .10	0,2367	0,0893	0,0058	0,0012	3	3
**Spot 8**	0,2300	0,2400	0,2400	0,0900	0,0880	0,0900	0	1	p < .10	0,2367	0,0893	0,0058	0,0012	3	3
**Spot 9**	0,5400	0,5600	0,5500	0,1000	0,1200	0,1050	0	1	p < .10	0,5500	0,1083	0,0100	0,0104	3	3
**Spot 10**	0,1200	0,1400	0,1300	0,0500	0,0500	0,0495	0	1	p < .10	0,1300	0,0498	0,0100	0,0003	3	3
**Spot 11**	0,1200	0,1400	0,1300	0,0500	0,0500	0,0500	0	1	p < .10	0,1300	0,0500	0,0100	0,0000	3	3
**Spot 12**	0,1100	0,1300	0,1200	0,0550	0,0500	0,0500	0	1	p < .10	0,1200	0,0517	0,0100	0,0029	3	3
**Spot 13**	0,1200	0,1200	0,1200	0,0600	0,0500	0,0600	0	1	p < .10	0,1200	0,0567	0,0000	0,0058	3	3
**Spot 14**	0,0700	0,0600	0,0700	0,0200	0,0400	0,0300	0	1	p < .10	0,0667	0,0300	0,0058	0,0100	3	3
**Spot 15**	0,0800	0,0750	0,0785	0,0340	0,0370	0,0360	0	1	p < .10	0,0778	0,0357	0,0026	0,0015	3	3
**Spot 16**	0,0900	0,0700	0,0810	0,0200	0,0310	0,0300	0	1	p < .10	0,0803	0,0270	0,0100	0,0061	3	3
**Spot 17**	0,0900	0,1000	0,1050	0,4500	0,5600	0,5100	-1	0	p < .10	0,0983	0,5067	0,0076	0,0551	3	3
**Spot 18**	0,0900	0,1100	0,1000	0,2500	0,2000	0,2200	-1	0	p < .10	0,1000	0,2233	0,0100	0,0252	3	3
**Spot 19**	0,1000	0,1200	0,1050	0,2100	0,2500	0,2300	-1	0	p < .10	0,1083	0,2300	0,0104	0,0200	3	3
**Spot 20**	0,2000	0,1900	0,2000	0,4000	0,4500	0,4300	-1	0	p < .10	0,1967	0,4267	0,0058	0,0252	3	3
**Spot 21**	0,1800	0,1700	0,1800	0,4000	0,5000	0,4500	-1	0	p < .10	0,1767	0,4500	0,0058	0,0500	3	3
**Spot 22**	0,9000	0,8000	0,8500	2,1000	1,9500	2,0000	-1	0	p < .10	0,8500	2,0167	0,0500	0,0764	3	3
**Spot 23**	0,1000	0,0800	0,0900	0,2200	0,2300	0,2300	-1	0	p < .10	0,0900	0,2267	0,0100	0,0058	3	3
