# Supplementary material for: Alterations in Kernel Proteome after Infection with Fusarium culmorum in Two Triticale Cultivars with Contrasting Resistance to Fusarium Head Blight
Source: Front Plant Sci. 2016 Aug 17;7:1217. doi: 10.3389/fpls.2016.01217 (PMC4987376; doi:10.3389/fpls.2016.01217)
Supplement: Supplementary file 3 [file Image2.pdf]

“Alterations in kernel proteome after infection with *Fusarium culmorum* in two triticale cultivars with contrasting resistance to *Fusarium* head blight”

Dawid Perlikowski[1#], Halina Wiśniewska[1#], Joanna Kaczmarek[1], Tomasz Góral[2], Piotr Ochodzki[2], Michał Kwiatek[1], Maciej Majka[1], Adam Augustyniak[1], Arkadiusz Kosmala[1*]

[1] Institute of Plant Genetics, Polish Academy of Sciences, Strzeszynska 34, 60-479 Poznan, Poland; [2] Plant Breeding and Acclimatization Institute - National Research Institute, Radzikow, 05-870 Blonie, Poland; [#] these authors participated equally in the research; akos@igr.poznan.pl

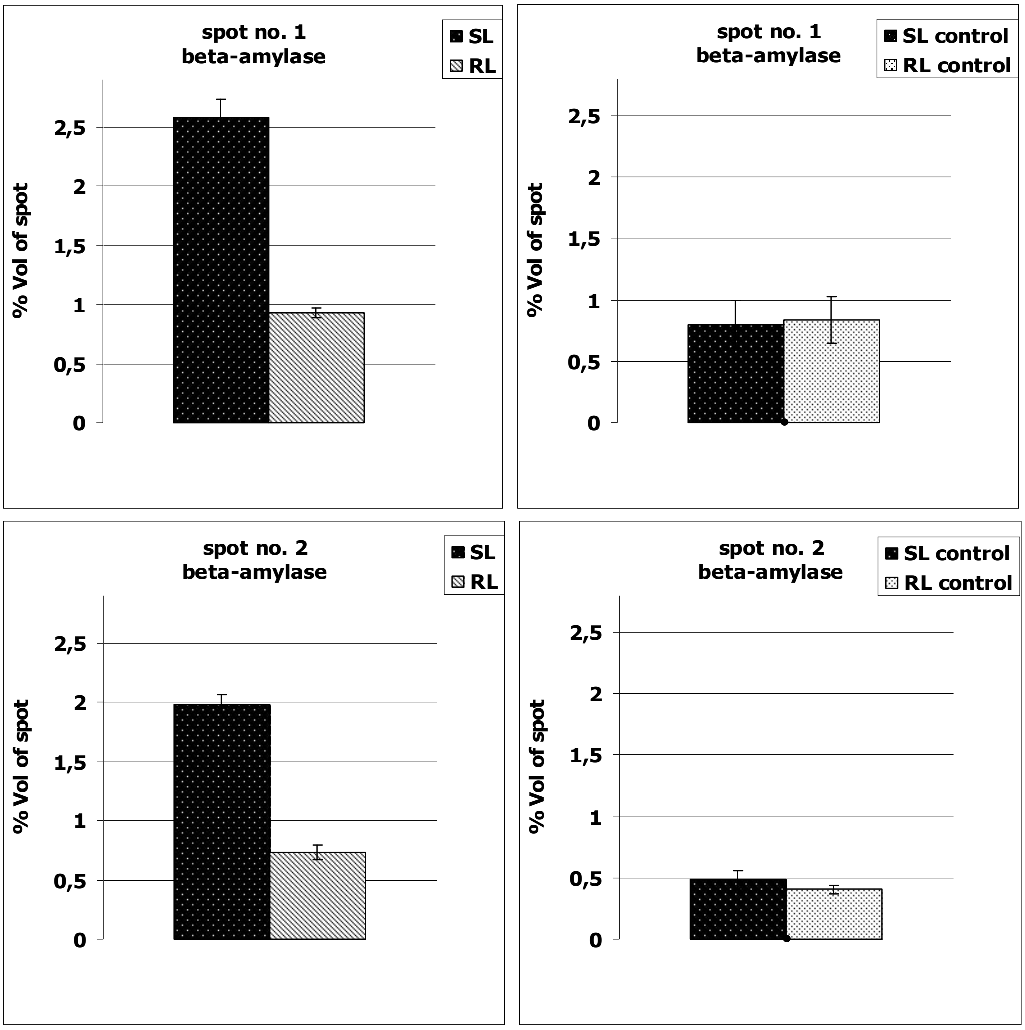

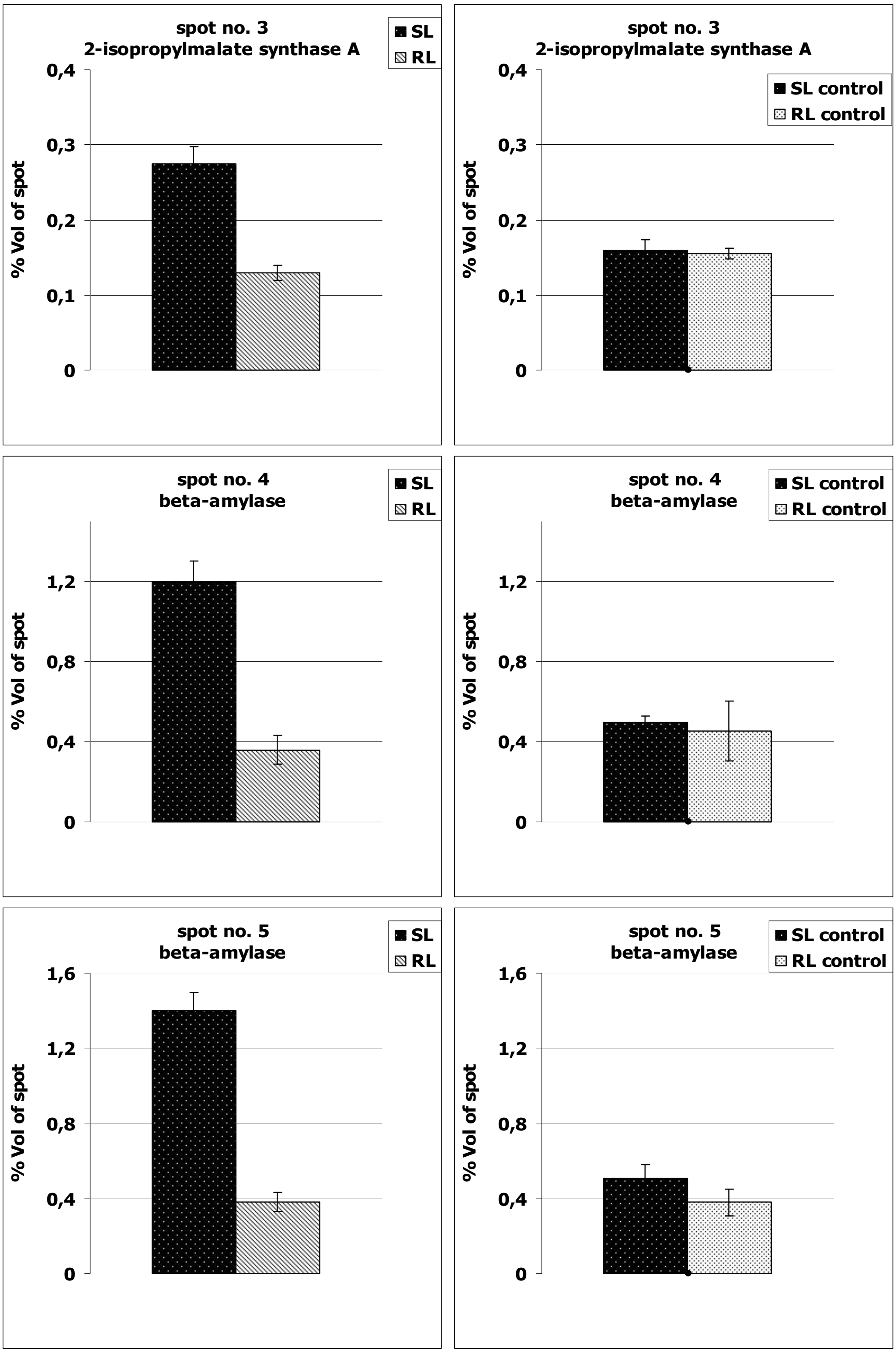
spot no. 3
2-isopropylmalate synthase A
SL
RL
% Vol of spot
0,4
0,3
0,2
0,1
0
spot no. 3
2-isopropylmalate synthase A
SL control
RL control
% Vol of spot
0,4
0,3
0,2
0,1
0
spot no. 4
beta-amylase
SL
RL
% Vol of spot
1,2
0,8
0,4
0
spot no. 4
beta-amylase
SL control
RL control
% Vol of spot
1,2
0,8
0,4
0
spot no. 5
beta-amylase
SL
RL
% Vol of spot
1,6
1,2
0,8
0,4
0
spot no. 5
beta-amylase
SL control
RL control
% Vol of spot
1,6
1,2
0,8
0,4
0

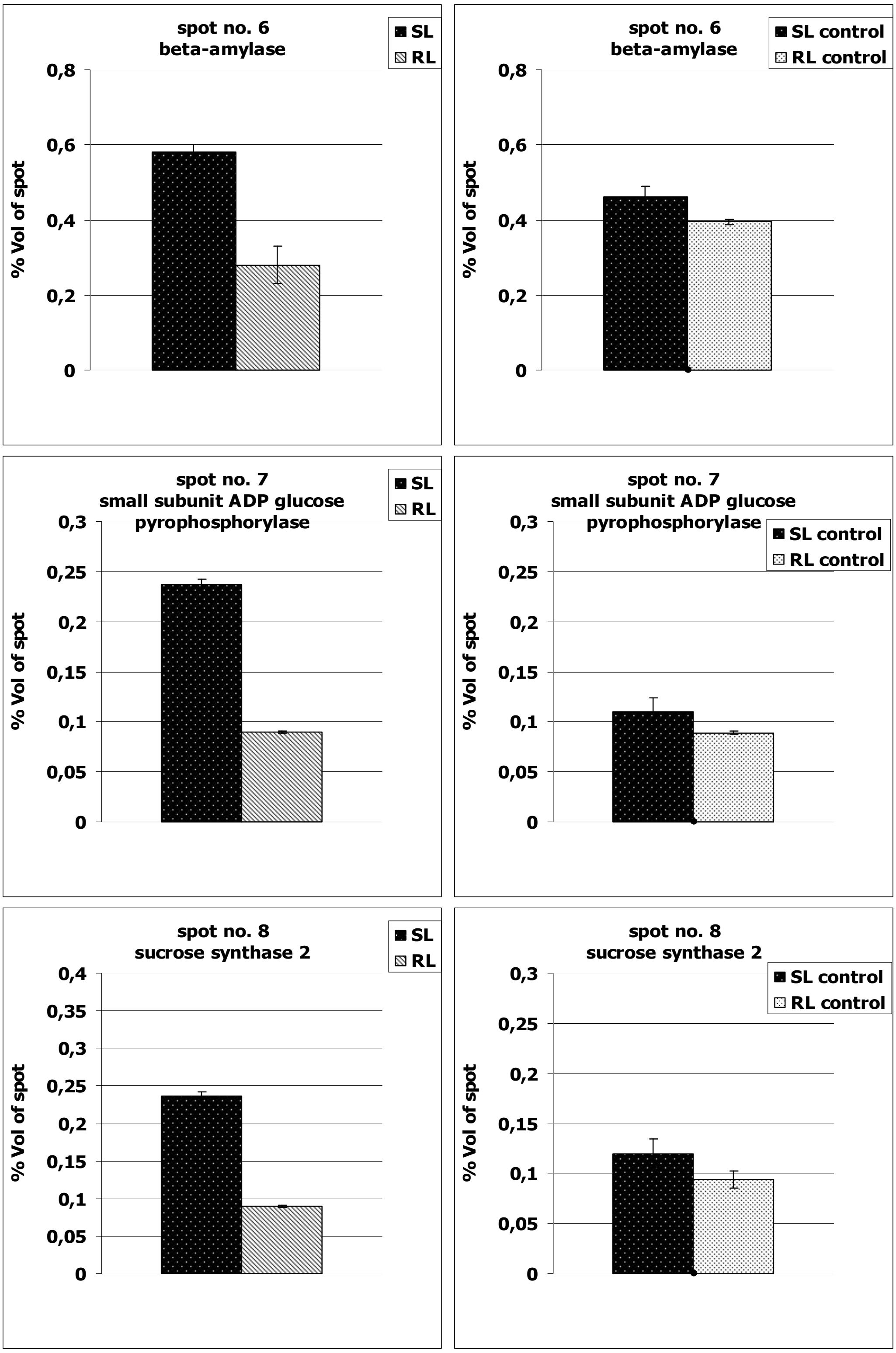
spot no. 6
beta-amylase
SL
RL
% Vol of spot
0,8
0,6
0,4
0,2
0
spot no. 6
beta-amylase
SL control
RL control
% Vol of spot
0,8
0,6
0,4
0,2
0
spot no. 7
small subunit ADP glucose pyrophosphorylase
SL
RL
% Vol of spot
0,3
0,25
0,2
0,15
0,1
0,05
0
spot no. 7
small subunit ADP glucose pyrophosphorylase
SL control
RL control
% Vol of spot
0,3
0,25
0,2
0,15
0,1
0,05
0
spot no. 8
sucrose synthase 2
SL
RL
% Vol of spot
0,4
0,35
0,3
0,25
0,2
0,15
0,1
0,05
0
spot no. 8
sucrose synthase 2
SL control
RL control
% Vol of spot
0,3
0,25
0,2
0,15
0,1
0,05
0

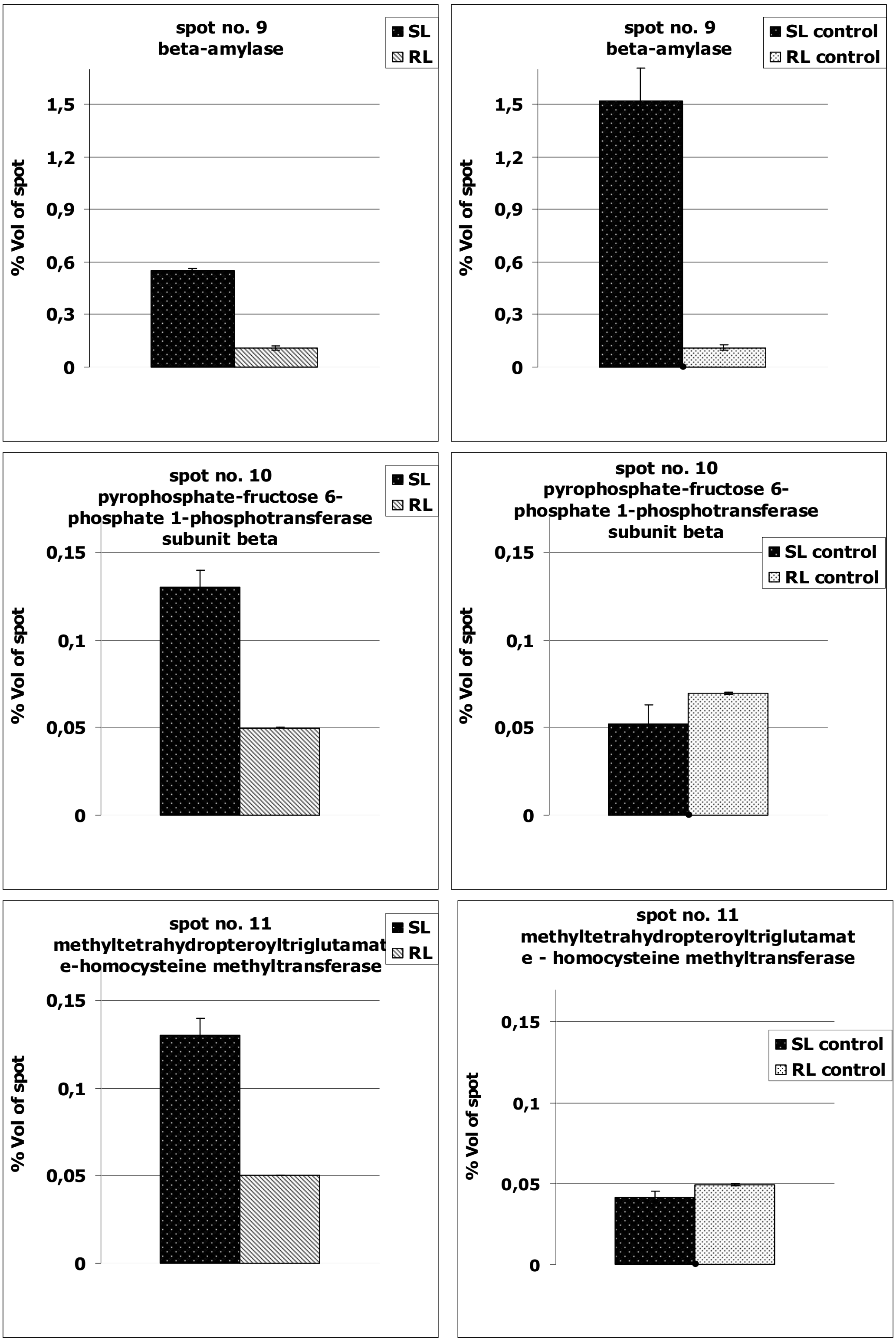
spot no. 9
beta-amylase
SL
RL
% Vol of spot
1,5
1,2
0,9
0,6
0,3
0
spot no. 9
beta-amylase
SL control
RL control
% Vol of spot
spot no. 10
pyrophosphate-fructose 6-phosphate 1-phosphotransferase subunit beta
SL
RL
% Vol of spot
0,15
0,1
0,05
0
spot no. 10
pyrophosphate-fructose 6-phosphate 1-phosphotransferase subunit beta
SL control
RL control
% Vol of spot
spot no. 11
methyltetrahydropteroyltriglutamate-homocysteine methyltransferase
SL
RL
% Vol of spot
spot no. 11
methyltetrahydropteroyltriglutamate - homocysteine methyltransferase
SL control
RL control
% Vol of spot

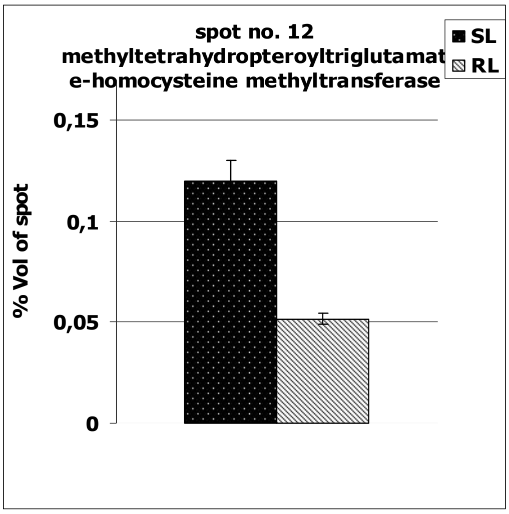
spot no. 12
methyltetrahydropteroyltriglutamate-homocysteine methyltransferase
SL
RL
% Vol of spot
0,15
0,1
0,05
0

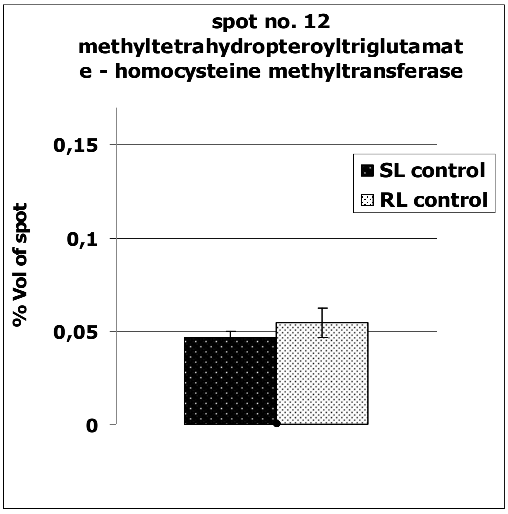
spot no. 12
methyltetrahydropteroyltriglutamate - homocysteine methyltransferase
SL control
RL control
% Vol of spot
0,15
0,1
0,05
0

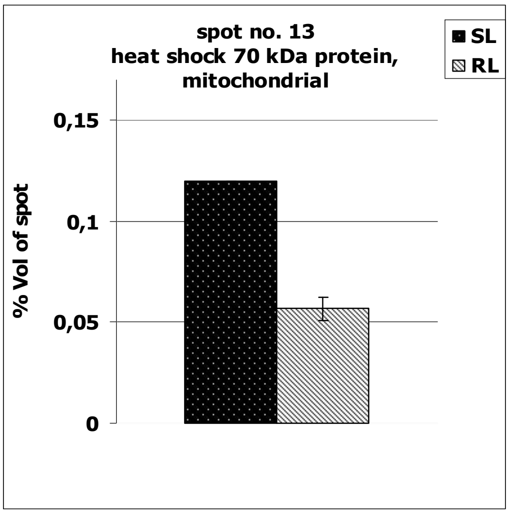
spot no. 13
heat shock 70 kDa protein, mitochondrial
SL
RL
% Vol of spot
0,15
0,1
0,05
0

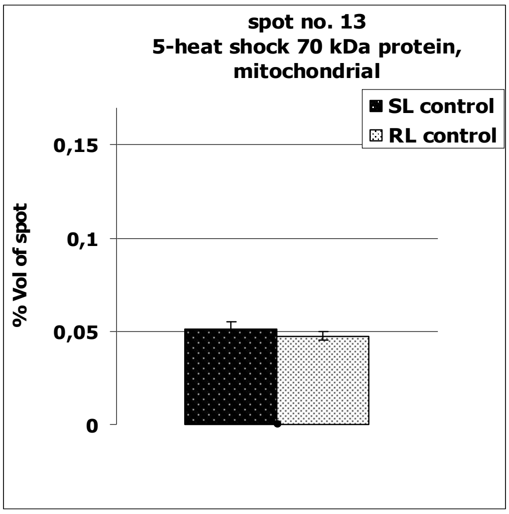
spot no. 13
5-heat shock 70 kDa protein, mitochondrial
SL control
RL control
% Vol of spot
0,15
0,1
0,05
0

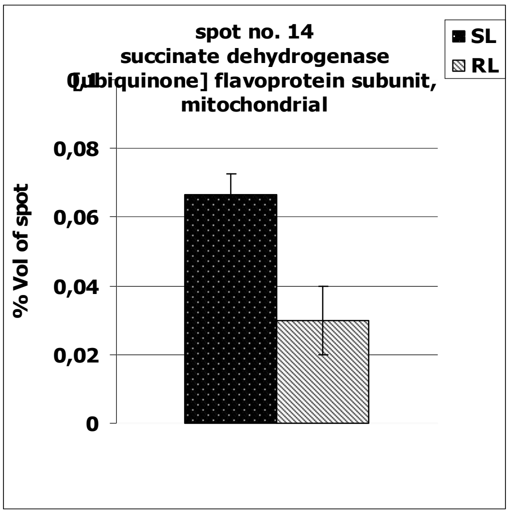
spot no. 14
succinate dehydrogenase [ubiquinone] flavoprotein subunit, mitochondrial
SL
RL
% Vol of spot
0,1
0,08
0,06
0,04
0,02
0

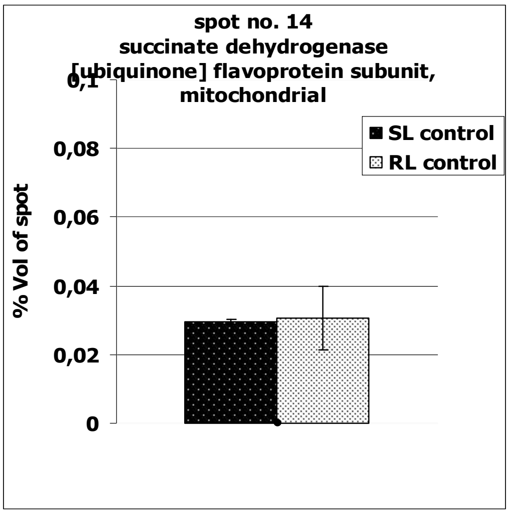
spot no. 14
succinate dehydrogenase [ubiquinone] flavoprotein subunit, mitochondrial
SL control
RL control
% Vol of spot
0,1
0,08
0,06
0,04
0,02
0

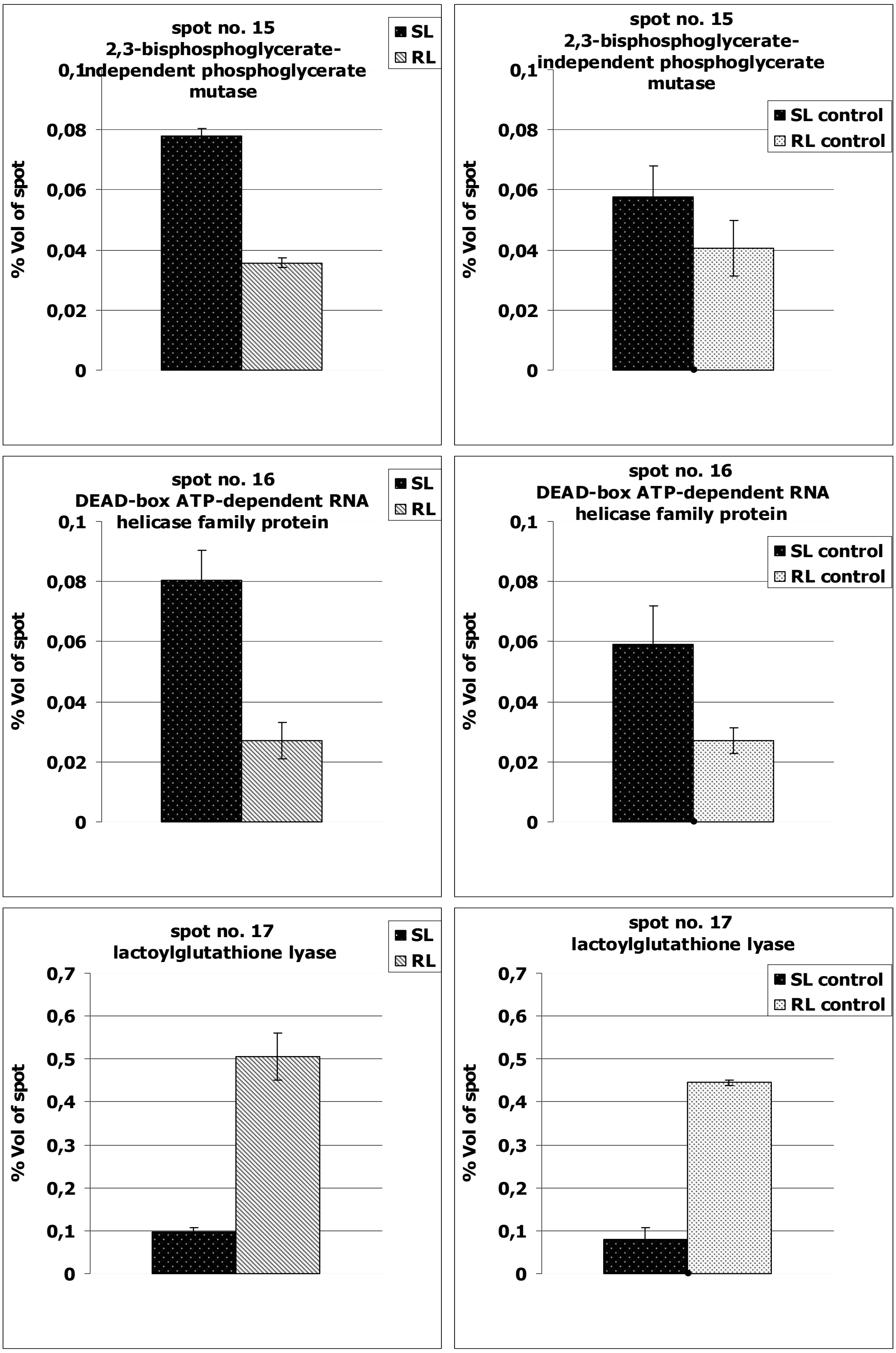
spot no. 15
2,3-bisphosphoglycerate-independent phosphoglycerate mutase
SL
RL
SL control
RL control
% Vol of spot
0
0,02
0,04
0,06
0,08
0,1
spot no. 16
DEAD-box ATP-dependent RNA helicase family protein
spot no. 17
lactoylglutathione lyase
0,1
0,2
0,3
0,4
0,5
0,6
0,7

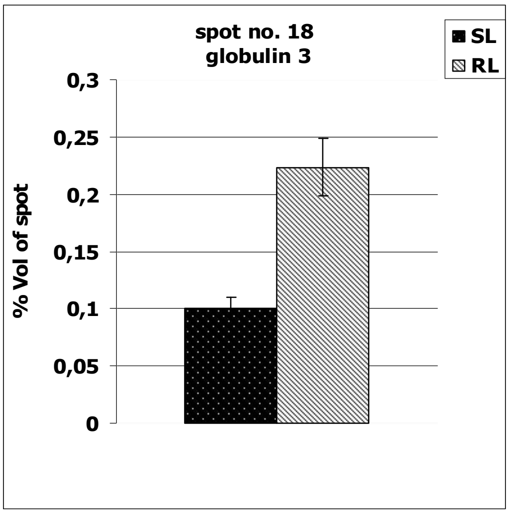
spot no. 18
globulin 3
SL
RL
% Vol of spot
0,3
0,25
0,2
0,15
0,1
0,05
0

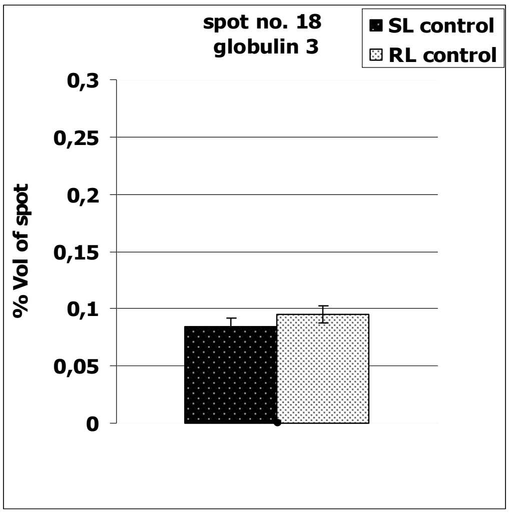
spot no. 18
globulin 3
SL control
RL control
% Vol of spot
0,3
0,25
0,2
0,15
0,1
0,05
0

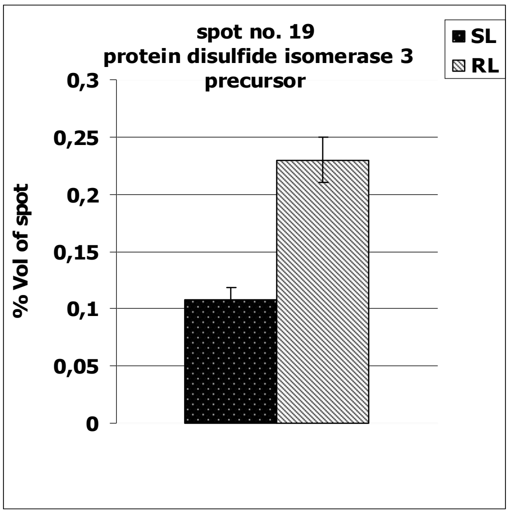
spot no. 19
protein disulfide isomerase 3
precursor
SL
RL
% Vol of spot
0,3
0,25
0,2
0,15
0,1
0,05
0

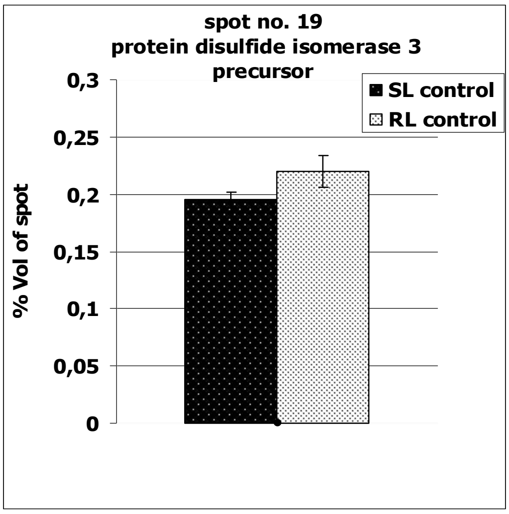
spot no. 19
protein disulfide isomerase 3
precursor
SL control
RL control
% Vol of spot
0,3
0,25
0,2
0,15
0,1
0,05
0

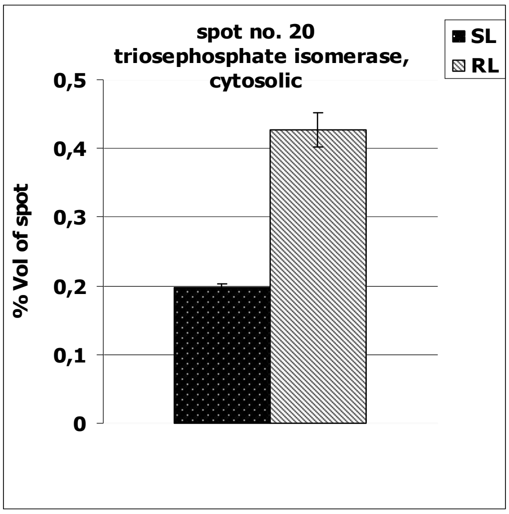
spot no. 20
triosephosphate isomerase,
cytosolic
SL
RL
% Vol of spot
0,5
0,4
0,3
0,2
0,1
0

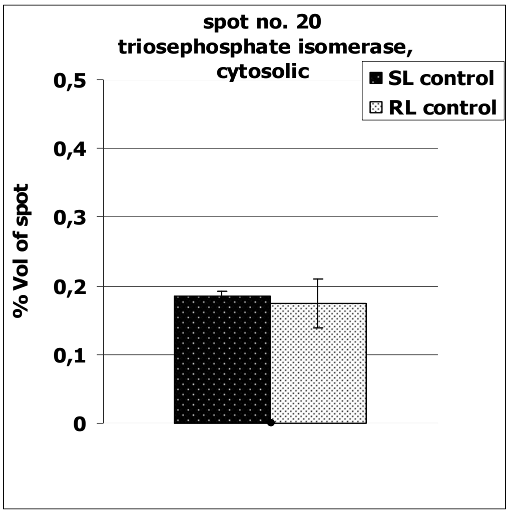
spot no. 20
triosephosphate isomerase,
cytosolic
SL control
RL control
% Vol of spot
0,5
0,4
0,3
0,2
0,1
0

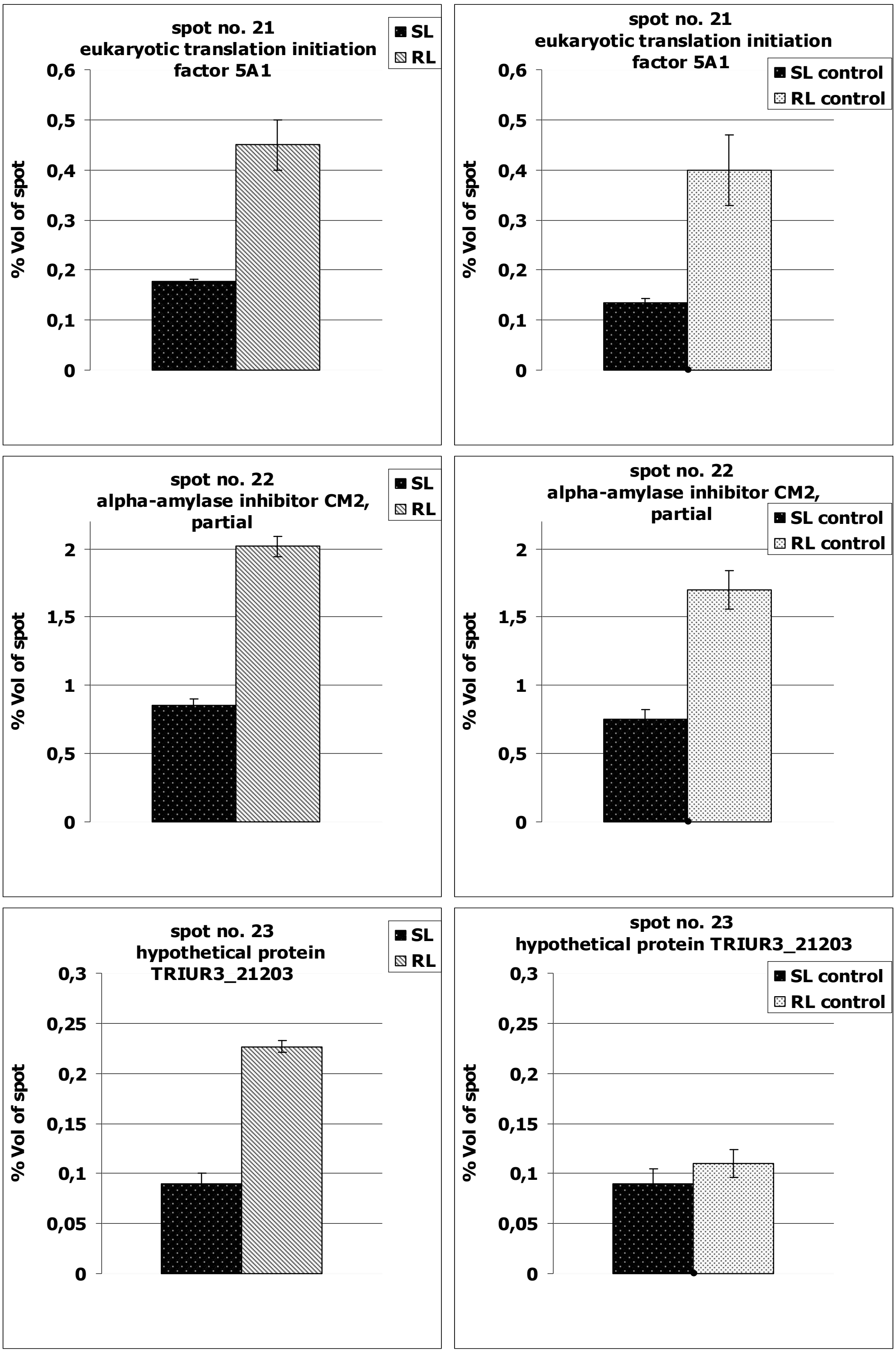

Figure S2 Comparison of selected kernel protein abundance after *Fusarium culmorum* infection and in the control conditions in the triticale SL (line more susceptible to *Fusarium* head blight) and the RL (line more resistant to *Fusarium* head blight). Spot numbering is the same as in Fig. 2 and 3. The means of %Vol (normalized volume) of spot (three biological replicates) and standard deviation bars after infection, are shown. The significance of

differences between the RL and SL after infection was assessed using Kolmogorov-Smirnov Test (three biological replicates, each one with two technical replicates used as means). In the control conditions the means of %Vol of spot (two technical replicates) and standard deviation bars are shown. The detail quantitative calculations and statistics are indicated in the Figure S1.
