# Supplementary material for: Alterations in Kernel Proteome after Infection with Fusarium culmorum in Two Triticale Cultivars with Contrasting Resistance to Fusarium Head Blight
Source: Front Plant Sci. 2016 Aug 17;7:1217. doi: 10.3389/fpls.2016.01217 (PMC4987376; doi:10.3389/fpls.2016.01217)
Supplement: Supplementary file 4 [file Image3.PDF]

“Alterations in kernel proteome after infection with *Fusarium culmorum* in two triticale cultivars with contrasting resistance to *Fusarium* head blight”

Dawid Perlikowski[1#], Halina Wiśniewska[1#], Joanna Kaczmarek[1], Tomasz Góral[2], Piotr Ochodzki[2], Michał Kwiatek[1], Maciej Majka[1], Adam Augustyniak[1], Arkadiusz Kosmala[1*]

[1] Institute of Plant Genetics, Polish Academy of Sciences, Strzeszynska 34, 60-479 Poznan, Poland; [2] Plant Breeding and Acclimatization Institute - National Research Institute, Radzikow, 05-870 Blonie, Poland; [#] these authors participated equally in the research; akos@igr.poznan.pl

Spot 1. beta-amylase

1 MEASVQQGNY VQVYVMLPLD AVSVNNRFEK GDELREQLKR LVEAGVDGVM
51 VDVWWGLVEG KGPR**AYDWSA YKQLFQLVHE AGLK**LKAIMS FHQCGGNVGD
101 VVNIPIPQWV R**NVGASDPDI FYTDQHGTRN IEYLTLGVDD QPLFHGRSAV**
151 **QMYTDYMASF R**DNMKEFLDA GVIVDIEVGL GPAGELRYPS YPQSHGWSFP
201 GIGEFICYDK **YLQADFKAAA AMVGHPEWEF PR**DAGQYNDA PQRTR**FFVDN**
251 **GTYLTEQGR**F FLAWYSNNLI KHGDK**ILDEA NK**VFLGHRVQ LAIK**ISGIHW**
301 **WYK**VPSHAAE ITAGYYNLHD R**DGYRPIAR**M LKRHR**ASLNF TCAEMR**DSEQ
351 SSQAMSAPEE LVQQVLSAGW R**EGLNMACEN ALPRYDPTAY NTILR**NARPH
401 GINK**SGPPEH KLFGFTYLRL SNQLVEGQNY VNFK**TFVDR**M HANLPHDPCV**
451 **DPVAPLQRSG PELTIEMILQ AAQPK**LDPFP FEEHTDLPVQ GLGGIGGDVE
501 GPSGGMGGEV QDPTGSMGGE LPATV

Spot 2. beta-amylase

1 MEASVQQGNY VQVYVMLPLD AVSVNNRFEK GDELREQLKR LVEAGVDGVM
51 VDVWWGLVEG KGPR**AYDWSA YKQLFQLVHE AGLK**LKAIMS FHQCGGNVGD
101 VVNIPIPQWV R**NVGASDPDI FYTDQHGTRN IEYLTLGVDD QPLFHGRSAV**
151 **QMYTDYMASF R**DNMKEFLDA GVIVDIEVGL GPAGELRYPS YPQSHGWSFP
201 GIGEFICYDK **YLQADFKAAA AMVGHPEWEF PR**DAGQYNDA PQRTR**FFVDN**
251 **GTYLTEQGR**F FLAWYSNNLI KHGDK**ILDEA NK**VFLGHRVQ LAIK**ISGIHW**
301 **WYK**VPSHAAE ITAGYYNLHD R**DGYRPIAR**M LKRHR**ASLNF TCAEMR**DSEQ
351 SSQAMSAPEE LVQQVLSAGW R**EGLNMACEN ALPRYDPTAY NTILR**NARPH
401 GINKSGPPEH K**LFGFTYLRL SNQLVEGQNY VNFK**TFVDRM HANLPHDPCV
451 DPVAPLQRSG PELTIEMILQ AAQPKLDPFP FEEHTDLPVQ GLGGIGGDVE
501 GPSGGMGGEV QDPTGSMGGE LPATV

Spot 3. 2-isopropylmalate synthase A

1 MASSSAKPYC CSSLNPAPSN AIARRAALSA LPAAKPRRFS HGLAAVAANP
51 RASRSAVLRP VRACLAAPR**R PEYVPDR**IDD PNYVR**IFDTT LRDGEQSPGA**
101 **TMTSAEK**LVV ARQLAR**LGVD IIEAGFPASS PDDLDAVRSI AIEVGNTPVG**
151 **EDGHVPVICG LSR**CNKR**DID AAWEAVR**HAR KPRIHTFIAT SEIHMQHKLR
201 **KTPDQVVAIA R**EMVAYAR**SL GCPDVEFSPE DAGR**SNREFL YHILEEVIK**A**
251 **GATTLNIPDT VGYTLPHEFG K**LIADIKANT PGIENAIIST HCQNDLGLAS
301 ANTLAGAYAG ARQLEVTING IGER**AGNASL EEVVMAIK**CR RELLGGLHTG
351 INSQHITMTS K**MVQEHSGLH VQPHKAIVGA NAFAHESGIH QDGMLK**FK**GT**
401 **YEIISPDDIG LTRANEFGIV LGK**LSGRHAV RTK**LVELGYE INDK**EFEDFF
451 KRYKEVAEKK KRVTDEDIEA LLSDEIFQPK VIWSLGDVQA TCGTLGLSTA
501 TVK**LITIDGE EKIGCSVGTG PVDAAYKAVD QIIQIPTVLR EYSMTSVTEG**
551 **IDAIATTR**VV IAGDVSADKP ALTSNSNR**SF SGSGAAMDVV VSSVRAYLSA**
601 **LNKMSSYVGA VK**ASSEAPET ISVQTTE

Spot 4. beta-amylase

1 MEASAQQGNY VQVYVMLPLD IVSVNNR**FEK GDELR**GQLKR LVEAGVDGVM
51 VDVWWGLVEG KGPR**VYDWSA YKQLFELVHE AGLK**LQAIMS FHQCGGNVGD
101 VVNIPIPQWV R**NVGVSDPDI FYTDQHGTRN IEYLTLGVDD QPLFHGRSAV**
151 **QMYADYMASF R**DNMKEFLDA GLIVDIEVGL GPAGELRYPS YPQSHGWSFP
201 GIGEFICYDK **YLQADFKAAA AMVGHPEWEF PRDAGTYNDT PQR**TR**FFVDN**
251 **GTYLTEQGR**F FLAWYSNNLI KHGDK**ILDEA NK**VFLGHTVQ LAIKISGIHW
301 WYK**VPSHAAE VTAGYYNLHD RDGYRPIAR**M LKR**HHASLNF TCAEMRDSEQ**
351 **SSQAMSAPEE LVQQVLSAGW REGLNMACEN ALPRYDPTAY NTILR**NARPH
401 GINK**SGPPEH KLFGFTYLRL SNQLVEGQNY VNFK**TFVDRM HANLPHDPCV
451 DPVAPLQR**SG PELTIEMILQ AAQPK**LEPFP FEEHTDLPVQ GLGGIGGGEV
501 EDPTGGMGGE VQQDPTGGMG GEVEDPTGGM GGELPPTV

Spot 5. beta-amylase

1 MEASVQQGNY VQVYVMLPLD AVSVNNR**FEK GDELR**EQLKR LVEAGVDGVM
51 VDVWWGLVEG KGPR**AYDWSA YKQLFQLVHE AGLK**LK**AIMS FHQCGGNVGD**
101 **VVNIPIPQWV RNVGASDPDI FYTDQHGTRN IEYLTLGVDD QPLFHGRSAV**
151 **QMYTDYMASF R**DNMKEFLDA GVIVDIEVGL GPAGELRYPS YPQSHGWSFP
201 GIGEFICYDK **YLQADFKAAA AMVGHPEWEF PR**DAGQYNDA PQRTR**FFVDN**
251 **GTYLTEQGR**F FLAWYSNNLI KHGDK**ILDEA NK**VFLGHRVQ LAIK**ISGIHW**
301 **WYKVPSHAAE ITAGYYNLHD RDGYRPIAR**M LKRHR**ASLNF TCAEMR**DSEQ
351 SSQAMSAPEE LVQQVLSAGW R**EGLNMACEN ALPRYDPTAY NTILR**NARPH
401 GINK**SGPPEH KLFGFTYLRL SNQLVEGQNY VNFK**TFVDR**M HANLPHDPCV**
451 **DPVAPLQRSG PELTIEMILQ AAQPK**LDPFP FEEHTDLPVQ GLGGIGGDVE
501 GPSGGMGGEV QDPTGSMGGE LPATV

Spot 6. beta-amylase

1 MEASAQQGNY VQVYVMLPLD IVSVNNR**FEK GDELR**GQLKR LVEAGVDGVM
51 VDVWWGLVEG KGPR**VYDWSA YKQLFELVHE AGLK**LQAIMS FHQCGGNVGD
101 VVNIPIPQWV R**NVGVSDPDI FYTDQHGTRN IEYLTLGVDD QPLFHGRSAV**
151 **QMYADYMASF R**DNMKEFLDA GLIVDIEVGL GPAGELRYPS YPQSHGWSFP
201 GIGEFICYDK **YLQADFKAAA AMVGHPEWEF PRDAGTYNDT PQR**TR**FFVDN**
251 **GTYLTEQGR**F FLAWYSNNLI KHGDK**ILDEA NK**VFLGHTVQ LAIKISGIHW
301 WYK**VPSHAAE VTAGYYNLHD RDGYRPIAR**M LKR**HHASLNF TCAEMR**DSEQ
351 SSQAMSAPEE LVQQVLSAGW R**EGLNMACEN ALPRYDPTAY NTILR**NARPH
401 GINKSGPPEH K**LFGFTYLR**L SNQLVEGQNY VNFKTFVDR**M HANLPHDPCV**
451 **DPVAPLQRSG PELTIEMILQ AAQPK**LEPFP FEEHTDLPVQ GLGGIGGGEV
501 EDPTGGMGGE VQQDPTGGMG GEVEDPTGGM GGELPPTV

Spot 7. small subunit ADP glucose pyrophosphorylase

1 MDVPLASK**TF PSPSPSK**REQ CNVDGHKSSS KHADLNPHAN DSVLGIILGG
51 GAGTR**LYPLT K**KRAKPAVPL GANYR**LIDIP VSNCLNSNIS KIYVLTQFNS**
101 **ASLNR**HLSRA YGSNIGGYKN EGFVEVLAAQ QSPDNPDWFQ GTADAVRQYL
151 WLFEEHNVME YLILAGDHLY RMDYEKFIQA HR**ETDADITV AALPMDEERA**
201 **TAFGLMK**IDE EGR**IIEFAEK PK**GEQLK**AMM VDTTILGLDD AR**AK**EMPYIA**
251 **SMGIYVISKH VMLQLLREQF PGANDFGSEV IPGATSTGMR** VQAYLYDGYW
301 EDIGTIEAFY NANLGITK**KP IPDFSFYDRS APIYTQPR**HL PPSK**VLDADV**
351 **TDSVIGEGCV IK**NCK**IHHSV VGLR**SCISEG AIIEDTLLMG ADYYETEADK
401 KLLAEK**GGIP IGIGK**NSHIK RAIIDKNAR**I GDNVMIINVD NVQEAARETD**
451 **GYFIKSGIVT VIK**DALLPSG TVI

Spot 8. sucrose synthase 2

1 MGETAGERAL SRIHSVRER**I GDSLSAHTNE LVAVFSR**LVN QGK**GMLQPHQ**
51 **ITAEYNAAIP EAER**EKLK**DT AFEDLLR**GAQ EAIVIPPWVA LAIRPRPGVW
101 EYVRVNVSEL GVEELSVAEY LQFKEQLANG SIDNNFVLEL DFEPFNASFP
151 RPSLSK**SIGN GVQFLNR**HLS SKLFHDK**ESM YPLLNFLR**AH NYK**GMTMMLN**
201 **DR**IR**SLGTLQ GALR**K**AETHL SGLPADTPYS EFHHRFQELG LEK**GWGDCAQ
251 RASETIHLLL DLLEAPDPSS LEKFLGTIPM VFNVVILSPH GYFAQANVLG
301 YPDTGGQVVY ILDQVR**AMEN EMLLR**IK**QQG LDITPKILIV TRLLPDAHGT**
351 **TCGQR**LEK**VL GTEHTHILR**V PFK**TEDGIVR** KWISRFEVWP YLEAYTDDVA
401 HEIAGELQAT PDLIIGNYSD GNLVACLLAH KLGVTHCTIA HALEK**TKYPN**
451 **SDLYWK**KFED HYHFSCQFTA DLIAMNHADF IITSTFQEIA GNK**DTVGQYE**
501 **SHMAFTMPGL YR**VVHGIDVF DPK**FNIVSPG ADMSIYFPYT EQQK**RLTSLH
551 TEIEELLFSD IENAEHKFVL KDKK**KPIIFS MAR**LDRVK**NM TGLVEMYGR**N
601 PR**LQELVNLV VVCGDHGK**VS K**DKEEQAEFK** K**MFDLIEQYN LIGHIR**WISA
651 QMNRVRNGEL YR**YICDMK**GA FVQPAFYEAF GLTVIEAMTC GLPTFATAYG
701 GPAEIIVNGV SGYHIDPYQN DK**ASALLVDF FGKCKEDPSH WNK**ISQGGLQ
751 RIEEKYTWKL YSER**LMTLSG VYGFWKYVSN LDR**RETR**RYL EMLYALK**YRK
801 **MAETVPLAVE GETSGK**

Spot 9. beta-amylase
1 MEASAQQGNY VQVYVMLPLD IVSVNNRFEK GDELRGQLKR LVEAGVDGVM
51 VDVWWGLVEG KGPR**VYDWSA YK**QLFELVHE AGLKLQAIMS FHQCGGNVGD
101 VVNIPIPQWV RNVGVSDPDI FYTDQHGTR**N IEYLTLGVDD QPLFHGRSAV**
151 **QMYADYMASF R**DNMKEFLDA GLIVDIEVGL GPAGELRYPS YPQSHGWSFP
201 GIGEFICYDK **YLQADFKAAA AMVGHPEWEF PR**DAGTYNDT PQRTRFFVDN
251 GTYLTEQGRF FLAWYSNNLI KHGDK**ILDEA NK**VFLGHTVQ LAIKISGIHW
301 WYKVPSHAAE VTAGYYNLHD R**DGYRPIAR**M LKRHHASLNF TCAEMRDSEQ
351 SSQAMSAPEE LVQQVLSAGW REGLNMACEN ALPR**YDPTAY NTILR**NARPH
401 GINKSGPPEH KLFGFTYLR**L SNQLVEGQNY VNFK**TFVDRM HANLPHDPCV
451 DPVAPLQRSG PELTIEMILQ AAQPKLEPFP FEEHTDLPVQ GLGGIGGGEV
501 EDPTGGMGGE VQQDPTGGMG GEVEDPTGGM GGELPPTV

Spot 10. pyrophosphate--fructose 6-phosphate 1-phosphotransferase subunit beta
1 MRWREDDKMV KMRRLAHEVT SFIIHRCAIV VTHLSDLIKH KKKKKENNLH
51 VSIKHDMQIL RYPTHIQHTY SESWQTLKGP FDGESSSWKA LQYHYLQERA
101 K**GSTMYGFKG GPAGVMK**GK**Y VELTTDFVYP YRNQGGFDMI CSGRDKIETP**
151 **EQFQQAEDTV NR**LDLDGLVV IGGDDSNTNA CLLGEYFRGR NLKTRVIGCP
201 K**TIDGDLK**CK EVPTSFGFDT ACK**IYSEMIG NVMTDAR**STG KYYHFVRLMG
251 RAASHITLEC ALQTHPNVAL IGEEVAEKKE TLK**NVTDYIT DVVCK**RAELG
301 YNYGVVLIPE GLIDFIPEIQ KLIAELNEIL AHDVVDEAGA WKSKLEPASR
351 **ELFDFLPKTI QEQLLLERDP HGNVQVAK**IE TEKMLIAMVE TELEKRRAAG
401 K**YSAHFRGQS HFFGYEGR**CG LPTNFDSSYC YALGYGAGAL LQFGKTGLIS
451 SVGNLAAPVE EWTVGGTALT ALMDVERRHG KNKPVIK**KAM VELDAAPFKK**
501 FASLR**DEWAS K**NRYISPGPI QFSGPGSDAS NHTLMLELGA EI

Spot 11. 5-methyltetrahydropteroyltriglutamate-homocysteine methyltransferase
1 MASHIVGYPR MGPKRELK**FA LESFWDGKSS AEDLEK**VATD LRASIWK**QMA**
51 **DAGIKYIPSN TFSYYDQVLD TTAMLGAVPD R**YSWTGGEIN LSTYFSMAR**G**
101 **NATVPAMEMT K**WFDTNYHFI VPELAPSTKF SYSSHKAINE YKEAKALGVD
151 TVPVLVGPVS YLLLSKAAKG VEKSFSPLSL LSSILPVYKE VIAELK**AAGA**
201 **SWIQFDEPTL VK**DLESHQLS AFSAAYAELE SALSGLNVLV ETYFADVPAD
251 SYK**TLTSLSS VTAYGFDLER** GTK**TLELVK**S GFPAGK**YLFA GVVDGR**NIWA
301 DDLAASLATL QSLEAVVGKD K**LVVSTSCSL MHTAVDLVNE TKLDDEIKSW**
351 **LAFAAQKVVE VNALAK**ALAG QK**DEAYFAAN AAALASR**RSS PRVTNEEVQK
401 AATALKGSDH RRATTVSARL DAQQKKLNLP VLPTTTIGSF PQTVELRRVR

451 REYKAK**KISE EEYTNAIKEE ISK**VVKIQEE LDIDVLVHGE PERNDMVEYF
501 GEQLSGFTFT ANGWVQSYGS RCVKPPIIYG DVSRPNPMTV FWSKMAQSMT
551 ARPMKGMLTG PVTILNWSFV RNDQPRFETC YQIALAIK**KE VEDLEAGGIQ**
601 **VIQIDEAALR** EGLPLRKSEH AFYLDWAVHS FRITNCGVQD TTQIHTHMCY
651 SNFNDIIQSI INMDADVITI ENSRSDEK**LL SVFREGVVYG AGIGPGVYDI**
701 **HSPRIPSKEE IADR**VNKMLA VLDTNILWVN PDCGLKTRK**Y AEVKPALTNM**
751 **VEAAK**QIRAE LAKAQ

Spot 12. 5-methyltetrahydropteroyltriglutamate-homocysteine methyltransferase

1 MASHIVGYPR MGPKRELK**FA LESFWDGKSS AEDLEK**VATD LRASIWK**QMA**
51 **DAGIK**YIPSN TFSYYDQVLD TTAMLGAVPD RYSWTGGEIN LSTYFSMAR**G**
101 **NATVPAMEMT KWFDTNYHFI VPELAPSTK**F SYSSHKAINE YKEAK**ALGVD**
151 **TVPVLVGPVS YLLLSK**AAKG VEKSFSPLSL LSSILPVYKE VIAELK**AAGA**
201 **SWIQFDEPTL VK**DLESHQLS AFSAAYAELE SALSGLNVLV ETYFADVPAD
251 SYK**TLTSLSS VTAYGFDLER** GTK**TLELVK**S GFPAGK**YLFA GVVDGR**NIWA
301 DDLAASLATL QSLEAVVGKD K**LVVSTSCSL MHTAVDLVNE TKLDDEIKSW**
351 **LAFAAQKVVE VNALAK**ALAG QK**DEAYFAAN AAALASR**RSS PRVTNEEVQK
401 AATALKGSDH RRATTVSARL DAQQKKLNLP VLPTTTIGSF PQTVELRRVR
451 REYKAK**KISE EEYTNAIKEE ISK**VVKIQEE LDIDVLVHGE PERNDMVEYF
501 GEQLSGFTFT ANGWVQSYGS RCVKPPIIYG DVSRPNPMTV FWSKMAQSMT
551 ARPMKGMLTG PVTILNWSFV RNDQPRFETC YQIALAIK**KE VEDLEAGGIQ**
601 **VIQIDEAALR** EGLPLRKSEH AFYLDWAVHS FRITNCGVQD TTQIHTHMCY
651 SNFNDIIQSI INMDADVITI ENSRSDEK**LL SVFREGVVYG AGIGPGVYDI**
701 **HSPRIPSKEE IADR**VNKMLA VLDTNILWVN PDCGLKTR**KY AEVKPALTNM**
751 **VEAAK**QIRAE LAKAQ

Spot 13. heat shock 70 kDa protein, mitochondrial

1 MALRSTADRC FGHHSPLALE HVQIKVVAVH RGNRHGCHIS KSRNKFNLHL
51 SANVLHVSLT IYFVPHSLTL AKPLGNEVIG IDLGTTNSCV SVMEGKNAK**V**
101 **IENSEGTRTT PSVVAFSQK**G ERLVGTPAKR **QAITNPQNTF FGTK**RMIGR**R**
151 **FDDPQTQK**EM KMVPYKIVK**A PNGDAWVETT DGKQYSPSQI GAFVLTKMKE**
201 **TAESYLGK**SI SK**AVITVPAY FNDAQR**QATK DAGR**IAGLDV QRIINEPTAA**
251 **ALSYGTNNK**E GLIAVFDLGG GTFDVSILEI SNGVFEVKAT NGDTFLGGED
301 FDNTLLEYLV SEYK**RSDNID LSKDR**LALQR LREAAEKAKI ELSSTAQTEI
351 NLPFITADAA GAKHLNITLT R**SKFESLVNG LIAR**TRDPCK NCLKDAGITT
401 K**EVDEVLLVG GMTR**VPK**VQE VVSEIFGK**AP SK**GVNPDEAV AMGAALQGGI**
451 **LR**GDVKELLL LDVTPLSLGI ETLGGIFTRL ITRNTTIPTK **KSQVFSTAAD**
501 **NQTQVGIR**VL QGEREMATDN K**LLGEFDLVG IPPAPR**GLPQ IEVTFDIDAN
551 GIVTVSAKDK ATAK**EQQITI RSSGGLSESE IEK**MVREAEL HSQKDQERKA
601 LIDIR**NTADT TIYSIEK**SLG EYRDK**IPAEV ATEIETAVAD LRAEMASDDI**
651 **EK**IKGKMEAA NKAVSK**IGEH MSGGGAAGGG AAGGGSQEGG SQGGGDQAPE**
701 **AEYEEVKK**

Spot 14. succinate dehydrogenase [ubiquinone] flavoprotein subunit, mitochondrial

1 MWRSRVSRGL REAKAAAAAA SRRFSTTSSY TVVDHTYDAV VVGAGGAGLR
51 **AAIGLSEHGF NTACITK**LFP TRSHTVAAQG GINAALGNMS EDDWRWHMYD
101 TVK**GSDWLGD QDAIQYMCR**E APK**AVIELEN YGLPFSR**TED GKIYQR**AFGG**
151 **QSLDFGK**GGQ AYRCACAADR **TGHAMLHTLY GQAMK**HNTQF FVEYFALDLI
201 MDK**EGTCQGI IALNMEDGTL HR**FR**STNTIL ATGGYGRAYF SATSAHTCTG**
251 **DGNAMVAR**AG LPLQDLEFVQ FHPTGIYGAG CLITEGSRGE GGILRNSEGE
301 RFMERYAPTA KDLASRDVVS R**SMTMEIR**EG RGVGPMKDHL YLHLNHLPPE
351 VLKERLPGIS ETAAIFAGVD VTKEPIPVLP TVHYNMGGIP TNYHGQVVDI
401 KGDNPDTIIP GLMAAGEAAC ASVHGETQKP LEKDAGEKTI AWLDKLR**NAN**

451 **GSLPTSK**IRL NMQR**IMQNNA AVFRTQETLT EGCELISEAQ K**SFHDVKLSD
501 RSLIWNSDLI ETIELENLLI NACITMHSAE ARQESRGAHA REDFKTR**DDD**
551 **KWMKHSLGYW EDEK**VRLEYR PVHMNTLDDE VETFPPKARV Y

Spot 15. 2,3-bisphosphoglycerate-independent phosphoglycerate mutase

1 MSPRQPAPSG PSNTRGRNIK LADPIVPNQA KAPEMATANW TLPDHPTLPK
51 GKTVAVVVLD GWGEASPDQY NCIHVAQTPV MDSLKNGAPE KWRLVK**AHGT**
101 **AVGLPSDDDM GNSEVGHNAL GAGRIFAQGA KLVDAALASG KIWEDEGFNY**
151 **IK**ESFDK**GTL HLIGLLSDGG VHSRLDQVQL IVK**GASERGA KRIR**LHILTD**
201 **GR**DVLDGSSV GFIETIEKDL AQLR**EQGVDA R**IASGGGR**MY VTMDRYENDW**
251 **SVVKRGWDAQ VLGEAPHKFQ NALEAVK**TLR AEPK**ANDQYL PPFVIVDESG**
301 **K**SVGPIVDGD AVVTFNFRAD RMVMLAK**ALE FPDFDKFDR**V RVPKIK**YAGM**
351 **LQYDGELK**LP SK**YLVSPPLI ERTSGEYLVK** NGVRTFACRQ VSILPLKTNM
401 GQVLGKIETV K**FGHVTFFWN GNRSGYFDET REEYVEIPSD SGITFNEQPK**
451 MK**ALEIAER**T RDAILSGKFD QVR**INLPNGD MVGHTGDIEA TVVACK**AADE
501 AVK**MVLDAVE QVGGIYLVTA DHGNAEDMVK** RNK**AGQPMLD K**SGSIQILTS
551 HTLQPVPVAI GGPGLHPGVR FRSDIQTPGL ANVAATVMNL HGFQAPADYE
601 TTLIEVVDK

Spot 16. putative DEAD-box ATP-dependent RNA helicase family protein

1 **MAPEGSQFDA K**HYDSKMQEL LSTGETEEFF TSYDEVFESF DDMGLQENLL
51 R**GIYAYGFEK PSAIQQR**GIV PFCK**GLDVIQ QAQSGTGK**TA TFCSGILQQL
101 DYGLVECQAL VLAPTR**ELAQ QIEK**VMR**ALG DYLGVKVHAC VGGTSVR**EDQ
151 RILGSGVHVV VGTPGR**VFDM LR**R**QSLRPDN IKMFVLDEAD EMLSR**GFKDQ
201 IYDIFQLLPA K**IQVGVFSAT MPPEALEITR** KFMNKPVRIL VK**RDELTLEG**
251 **IK**QFYVNVDK EDWKLDTLCD LYETLAITQS VIFVNTRR**KV DWLTDK**MRSR
301 **DHTVSATHGD MDQNTR**DIIM REFRSGSSR**V LITTDLLAR**G IDVQQVSLVI
351 NYDLPTQPEN YLHRIGRSGR FGRK**GVAINF VTR**DDER**MLF DIQK**FYNVLI
401 EELPANVADL L

Spot 17. lactoylglutathione lyase

1 MATGSDAGK**S AEAVLEWPKQ DK**KRMLHAVY RVGDLDRTIK **CYTECFGMK**L
51 LRK**RDVPEEK** YTNAFLGYGP EDTNFALELT YNYGVDKYDI GAGFGHFAIA
101 NEDVYK**LSET IKSSDCCKIT REPGPVK**GGS TVIAFAQDPD GYMFELIQR**G**
151 **PTPEPLCQVM LR**VGDLDR**AI MFYEK**ALGMK LLRK**KDVPQY KYTIAMMGYA**
201 **EEDKTTVLEL TYNYGVTEYN KGNAYAQVAI GTDDVYKSAE AVELVTK**ELG
251 GKILR**QPGPL PGLNTKITSF LDPDGWKVVL VDHADFLK**EL H

Spot 18. globulin 3

1 MATRARVTIP LLFLLGTSLL FAAAVSASHD EEEDRRGGRS LQQCVQRCQQ
51 DRPRYSHARC VQECRDDQQQ HGRHEQEEQG RGHGRHGEGE REEEQGRGRG
101 RHGQGEREEE QGRGRGRRGE GERDEEHGDG RRPYVFGPRS FRRIIRSDHG
151 FVKALRPFDE VSRLLRGIRN YRVAIMEVNP RAFVVPGLTD ADGVGYVAQG
201 EGVLTVIENG EKRSYTVRQG DVIVAPAGSI MHLANTDGRR KLVIAKILHT
251 ISVPGKFQYF SAKPLLASLS KRVLTAALKT SDERLGSLLG SRQGKEEEEK
301 SISIVRASEE QLRELRRQAS EGDQGHHWPL PPFRGDSRDT FNLLEQRPKI
351 ANRHGR**LYEA DAR**SFHALAQ HDVRVAVANI TPGSMTAPYL NTQSFKLAVV
401 LEGEGEVEIV CPHLGRDSER REQEHGKGRW RSEEEEDDRR QQRR**RGSGSE**
451 **SEEEQDQQRY ETVR**ARVSRG SAFVVPPGHP VVEIASSRGS SNLQVVCFEI
501 NAERNERVWL AGRNNVIAKL DDPAQELTFG RPAR**EVQEVF R**AKDQQDEGF
551 VAGPEQQQEH ERGDRRRGDR GRGDEAVEAF LRMATAAL

Spot 19. protein disulfide isomerase 3 precursor

1 MAICKAWISL LLALAVVLSA PAARAEEAAA AAEEAAAPEA VLTLHADNFD
51 DAIAKHPFIL VEFYAPWCGH CK**SLAPEYEK AAQLLSKHDP AIVLAK**VDAN
101 DEKNKPLAGK **YEVQGFPTLK** IFRNGGKNIQ EYKGPR**EAEG IVEYLKK**QVG
151 PASKEIK**APE DATYLEDGK**I HIVGVFTEFS GTEFTNFLEV AEKLRSDYDF
201 GHTVHANHLP R**GDAAVERPL VR**LFKPFDEL VVDSKDFDVS ALEK**FIDASS**
251 **TPK**VVTFDKN PDNHPYLLKF FQTNAPKAML FLNFSTGPFE SFK**SAYYGAV**
301 **EEFSGK**DVKF LIGDIEASQG AFQYFGLKED QAPLILIQDS DSKKFLKEQV
351 EAGQIVAWLK DYFDGKLTPF RKSEPIPEAN NEPVKVVVAD NVHDVVFKSG
401 KNVLIEFYAP WCGHCKKLAP ILDEAAATLQ SEEDVVIAKM DATANDVPSE
451 FDVQGYPTLY FVTPSGKKVS YEGGRTADEI VDYIKKNK**ET AGQAAAADTE**
501 **K**AAEPAATEP LKDEL

Spot 20. triosephosphate isomerase, cytosolic

1 MGRK**FFVGGN WK**CNGTVSQV ETIVNTLNAG QIASPDVVEV VVSPPYVFLP
51 TVKDKLRPEI QVAAQNCWVK KGGAFTGEVS AEMLVNLGIP WVILGHSER**R**
101 **SLLAESSEFV GEKVAYALAQ GLKVIACVGE TLEQREAGST MEVVAEQTK**A
151 IADKIKDWTN VVVAYEPVWA IGTGK**VASPA QAQEVHANLR** DWLK**TNVSPE**
201 **VAESTRIIYG GSVTGASCK**E LAAQPDVDGF LVGGASLKPE FIDIINAATV
251 KSA

Spot 21. eukaryotic translation initiation factor 5A1

1 MSDTDEHHFE SKADSGASK**T YPQQAGAIRK GGHIVIK**ARP CK**VVEVSTSK**
51 TGKHGHAKCH FVAIDIFNGK KLEDIVPSSH NCDVPHVDRQ DYQLIDITDD
101 GYVSLLTESG NTKDDLK**LPT DDVLLGQIK**T GFADGKDLIL SVMSAMGEEQ
151 ICAVKEIGGG K

Spot 22. putative alpha-amylase inhibitor CM2, partial

1 **TGPYCYPGMG LPSNPLEGCR EYVAQQTCGV GIVGSPVSTE PGNTPR**DRCC
51 KELYDASQHC WCEAVRYFIG R**TSDPNSGVL KDLPGCPR**EP QRDSAKVLVT
101 PGHCNVMTVH NTPYCLGLDI

Spot 23. hypothetical protein TRIUR3_21203

1 MASGAVRPK**L AYIVCYVEDV GKSAAFYAKA FNYSVR**RVDD SHK**WAELDTG**
51 **STTIAFTPR**H QR**ETDALTGE VQLPK**SPRER GPVEICFDYD DVDAAYRRGV
101 ENGAVPVSAP EQKNWGQKVG YVR**DCDGITV R**LGSHVRE

Fig. S3 Amino acid sequences of the identified proteins. In bold the amino acid sequence of peptides derived from triticale, which were successfully matched to the protein sequences present in the database, are indicated. Spot numbers and protein names are shown.
